# TurkerNeXtV2: An Innovative CNN Model for Knee Osteoarthritis Pressure Image Classification

**DOI:** 10.3390/diagnostics15192478

**Published:** 2025-09-27

**Authors:** Omer Esmez, Gulnihal Deniz, Furkan Bilek, Murat Gurger, Prabal Datta Barua, Sengul Dogan, Mehmet Baygin, Turker Tuncer

**Affiliations:** 1 Department of Orthopedics, Elazig Fethi Sekin City Hospital, Elazig 23280, Turkey; dresmezomer@gmail.com; 2 Department of Physiotherapy and Rehabilitation, Faculty of Health Sciences, Erzurum Technical University, Erzurum 25050, Turkey; gulnihal.deniz@erzurum.edu.tr; 3 Department of Gerontology, Fethiye Faculty of Health Sciences, Mugla Sitki Kocman University, Mugla 48000, Turkey; furkanbilek@mu.edu.tr; 4 Department of Orthopedics, Firat University Hospital, Firat University, Elazig 23119, Turkey; muratgurger@firat.edu.tr; 5 School of Business (Information System), University of Southern Queensland, Toowoomba, QLD 4350, Australia; prabal.barua@usq.edu.au; 6 Department of Digital Forensics Engineering, Technology Faculty, Firat University, Elazig 23119, Turkey; turkertuncer@firat.edu.tr; 7 Department of Computer Engineering, Erzurum Technical University, Erzurum 25050, Turkey; mehmet.baygin@erzurum.edu.tr

**Keywords:** TurkerNeXtV2, osteoarthritis detection, deep learning, pooling-based attention, biomedical image classification

## Abstract

**Background/Objectives:** Lightweight CNNs for medical imaging remain limited. We propose TurkerNeXtV2, a compact CNN that introduces two new blocks: a pooling-based attention with an inverted bottleneck (TNV2) and a hybrid downsampling module. These blocks improve stability and efficiency. The aim is to achieve transformer-level effectiveness while keeping the simplicity, low computational cost, and deployability of CNNs. **Methods:** The model was first pretrained on the Stable ImageNet-1k benchmark and then fine-tuned on a collected plantar-pressure OA dataset. We also evaluated the model on a public blood-cell image dataset. Performance was measured by accuracy, precision, recall, and F1-score. Inference time (images per second) was recorded on an RTX 5080 GPU. Grad-CAM was used for qualitative explainability. **Results:** During pretraining on Stable ImageNet-1k, the model reached a validation accuracy of 87.77%. On the OA test set, the model achieved 93.40% accuracy (95% CI: 91.3–95.2%) with balanced precision and recall above 90%. On the blood-cell dataset, the test accuracy was 98.52%. The average inference time was 0.0078 s per image (≈128.8 images/s), which is comparable to strong CNN baselines and faster than the transformer baselines tested under the same settings. **Conclusions:** TurkerNeXtV2 delivers high accuracy with low computational cost. The pooling-based attention (TNV2) and the hybrid downsampling enable a lightweight yet effective design. The model is suitable for real-time and clinical use. Future work will include multi-center validation and broader tests across imaging modalities.

## 1. Introduction

Osteoarthritis (OA) is a condition where joint cartilage breaks down over time, leading to structural and functional changes in the joint, including the synovium, menisci, ligaments, and bones [1]. OA is a widespread public health problem worldwide and, as of 2020, is reported to affect approximately 595 million people; this burden has increased significantly over the past thirty years [2]. The clinical picture is mostly characterized by joint pain that increases with activity, stiffness, and functional limitations [3]. Current guidelines recommend symptom-focused topical and oral NSAIDs and intra-articular corticosteroid injections in appropriate cases; however, there is currently no approved pharmacological treatment that modifies the disease [2]. Pressure-sensitive gait paths (e.g., GAITRite), plantar pressure platforms, and wearable IMU-based systems are widely used in the objective assessment of gait disorders accompanying OA, and their validity and reliability have been demonstrated in the literature [4]. For early diagnosis, soluble biomarkers in serum/urine/synovial fluid (e.g., Type II collagen degradation products uCTX-II/sCTX-II, COMP, PIIANP) and quantitative MRI-based imaging markers (e.g., T2, T1ρ, dGEMRIC) show promise. However, solubility markers have limitations in specificity, circadian and activity-related variability, and inter-laboratory standardization issues. Imaging markers face significant constraints in scanner/protocol variation, cost, and inconsistent relationships between symptoms and long-term progression [5]. Within the scope of regulatory initiatives, FNIH PROGRESS OA studies focus on imaging and the characterization of biochemical markers; however, there is still no single early diagnostic biomarker that has achieved widespread clinical acceptance [6]. In this context, digital gait markers and low-cost measurements such as plantar pressure distribution offer a complementary approach to identifying specific gait signatures associated with OA, and recent studies have shown that machine learning can distinguish OA-related plantar pressure changes [7].

In knee osteoarthritis (KOA), the cartilage in the knee joint gradually wears away, the underlying bone changes shape, and inflammation develops in the joint lining. These changes cause pain, stiffness, and difficulty moving. KOA is a major health issue globally, particularly for older adults, and significantly impacts the quality of life of those affected [8,9]. Early detection and monitoring are essential to slow disease progression and improve outcomes. Detecting OA early can reduce healthcare costs and enable timely treatments. However, traditional methods, such as X-rays and symptom assessments, often identify advanced-stage OA when irreversible damage has already occurred. In recent years, deep learning-based approaches, particularly convolutional neural networks (CNN). Transformer-based models, have achieved high success in medical image classification [10,11]. Transformer-based models, originally introduced for natural language processing, rely on the self-attention mechanism to capture long-range dependencies between input tokens. When adapted to vision tasks, such as in the Vision Transformer (ViT) [12] and Swin Transformer [13], they divide images into patches and model contextual relationships across the entire image. This global receptive field allows Transformers to extract complex structural and spatial features beyond the local receptive field of CNNs. Consequently, Transformer-based architectures achieve high accuracy in medical image analysis by leveraging their ability to detect subtle and spatially dispersed abnormalities. Their superior performance has been reported in diverse domains, including histopathology [14,15], MRI [10], and X-ray classification for osteoarthritis [16,17]. Their ability to capture global context and subtle patterns makes them particularly suitable for detecting early or small-scale pathological changes. Although the literature on plantar-pressure image analysis has increased recently [18], architectures explicitly designed for plantar- pressure image classification remain relatively few. Some studies have focused on segmentation or key-point detection using U-Net or related CNN-based segmentation networks [19]. Others use CNNs more generically, e.g., for classifying static or dynamic plantar pressure in diabetic foot or walking duration/shoe pressure tasks [20]. However, most existing models are adaptations of general-purpose CNNs rather than new-generation CNNs custom-built for plantar-pressure image features (e.g., zonal variances, load distribution, foot shape). TurkerNeXtV2 aims to fill this gap by providing a CNN architecture tailored for plantar pressure images, with modules designed to capture region-specific attention and lightweight inference suitable for portable or wearable application.

Traditional plantar pressure analysis methods rely on simple indices such as the arch index, footprint-based assessments, and basic spatiotemporal gait parameters (e.g., stance time, center-of-pressure trajectory) [21,22]. While clinically useful, these approaches often miss complex spatiotemporal pressure distribution patterns and fail to detect subtle load imbalances that emerge during the early stages of osteoarthritis [9,23]. As a result, important biomechanical cues linked to disease onset may be overlooked, which highlights the need for computerized and data-driven approaches capable of extracting detailed pressure signatures. Wearable systems with machine learning (ML) provide a practical way to analyze plantar pressure for early OA screening and monitoring [7]. Plantar pressure means how load is distributed under the foot. Pressure-sensitive platforms measure this distribution and reveal gait and load changes that indicate early joint degeneration [24]. Since the knee and hip joints are critical for movement, subtle shifts in foot pressure may appear even before symptoms develop. ML models excel at identifying complex patterns in large datasets and are increasingly used in clinical biomechanics [7]. However, most existing approaches depend on the adaptation of large pre-trained models. While this improves performance, it also increases computational cost and raises the risk of overfitting when the dataset is small. Transformer-based methods often achieve high accuracy, but their heavy design makes them unsuitable for lightweight or mobile devices. Despite these challenges, such models can still identify subtle patterns in plantar pressure data that traditional techniques may miss, thereby improving diagnostic accuracy [25]. Early diagnosis of OA remains a major challenge, since radiographic findings often emerge only in advanced stages [26]. By leveraging plantar-pressure patterns, TurkerNeXtV2 provides a computerized deep learning–based decision-support system that can detect subtle gait abnormalities associated with early cartilage degeneration. Such computerized approaches can complement conventional imaging and clinical assessment, enabling timely interventions and reducing disease progression [27]. In this respect, TurkerNeXtV2 not only achieves high accuracy on plantar-pressure images but also offers a low-cost, portable solution for early OA screening in clinical and rehabilitation settings. This study explores the potential of ML algorithms to classify early-stage OA using foot pressure distribution data. By leveraging biomechanical insights and computational tools, we aim to develop a practical, scalable diagnostic method for early OA detection, supporting preventive care and personalized rehabilitation strategies. In this paper, TurkerNeXtV2, a new lightweight, modular, and interpretable CNN architecture, is proposed to address these shortcomings. The model incorporates the innovative TNV2 block, developed by combining a pooling-based attention mechanism and inverted bottleneck blocks, and has been evaluated on a newly compiled osteoarthritis pressure image dataset. This study aims to contribute to the development of low-cost and portable solutions for clinical applications in early diagnosis.

### 1.1. Related Works

CNNs have achieved significant success in various image classification tasks, particularly in the biomedical domain [28]. Despite the recent surge in transformer-based architectures, lightweight CNN models remain highly relevant due to their efficiency, portability, and low computational cost. In the context of medical imaging, compact CNN architectures have been widely used for the classification of histopathological slides, blood cell smears, and other clinical imagery, offering real-time diagnostic assistance with minimal hardware requirements. Moreover, hybrid and modular architectures such as ViT [12] and MLP-Mixer [29] have inspired new directions in CNN design by enhancing feature reuse and improving learning dynamics. Vision Transformer introduced patch-based tokenization and global self-attention, which encouraged CNN architectures to adopt patchify stems, larger receptive fields, and attention-inspired modules. MLP-Mixer, on the other hand, demonstrated that purely feed-forward token-mixing and channel-mixing operations could rival convolutional approaches, highlighting the importance of modularity and separation of spatial and channel processing. These innovations motivated the development of hybrid CNN designs such as ConvNeXt, PoolFormer, and our proposed TurkerNeXtV2, which borrow the modularity of Transformers and Mixers while preserving the efficiency of convolution. In this section, we review recent studies that focus on lightweight CNNs, biomedical image classification, and hybrid CNN-transformer models. Related works that align with our research context are summarized in Table 1.

In recent years, various deep learning-based methods have been developed for the automatic diagnosis and severity classification of knee osteoarthritis (OA). Tiulpin et al. [30] achieved 66.71% accuracy and an AUC value of 0.93 in five-class OA severity classification using their Siamese CNN-based model developed on the MOST and OAI X-ray datasets. Guan et al. [31] proposed a model that predicts pain progression by combining clinical risk factors with YOLO (You Only Look Once) -based ROI (Region of Interest) detection and the EfficientNet CNN architecture, achieving an AUC (Area Under Curve) value of 80.7%. Abdullah and Rajasekaran [32] reported a classification accuracy of 98.9% on the Madurai dataset using a two-stage approach with Faster R-CNN and AlexNet. Wang et al. [33] performed segmentation and KL classification using YOLO-based knee joint detection and a Transformer-supported ResNet50 architecture. Hu et al. [34] predicted KOA progression using multimodal MRI data with a 3D DenseNet169-based model and achieved an AUC value of 77.5% over a 24-month period. Rani et al. [35] achieved 92.3% accuracy in binary classification using a 12-layer CNN architecture on the OAI dataset. Ahmed and Imran [36] achieved 99.13% accuracy in binary classification by examining pre-trained CNN models such as EfficientNetb7 and ensured the model’s explainability with Grad-CAM (Gradient-weighted Class Activation Mapping). Finally, Touahema et al. [37] presented the MedKnee software developed with Xception-based CNN and transfer learning, reporting over 97% accuracy on OAI and external datasets. Table 1 makes the progression from our earlier baselines to the current model explicit under a common subject-wise evaluation protocol. TurkerNeXt [45] is a lightweight ConvNeXt-inspired baseline without explicit attention, which limits spatial focus and increases sensitivity to noise. Attention TurkerNeXt [46] adds channel and spatial attention, improving saliency at the cost of extra computing power, while its downsampling remains average. In TurkerNeXtV2 we redesign the attention pathway to incorporate pooled attention inside an inverted bottleneck and adopt hybrid downsampling, achieving a better accuracy–efficiency balance. As a result, we observe consistent gains in Accuracy, Macro-F1, and AUC, together with more stable and clinically meaningful saliency maps. The following sections (Methods) detail the TNV2 block and training setup, and (Results) report head-to-head comparisons and ablations under the same protocol. When these studies (Table 1) are examined, it is observed that despite achieving high accuracy rates, most approaches continue to have shortcomings such as relying on heavy architectures, having high data requirements, and limited clinical portability. This situation clearly highlights the need for new-generation CNN architectures that are lightweight, modular, and applicable in clinical settings.

### 1.2. Literature Gaps

- Transformers dominate general computer vision. In KOA imaging, however, most works still use CNNs due to compute, data size, and deployment limits.
- Lightweight, task-specific CNNs for plantar-pressure images are scarce. Existing models target X-ray or MRI.
- Many studies rely on large pretrained backbones. This improves accuracy on small sets but risks overfitting and domain shift.
- Transformer-based KOA studies exist (e.g., Swin/ViT or plug-in modules) but show mixed gains, especially for early KL grades, and often lack external validation.
- Public plantar-pressure datasets are limited. Reproducible baselines and modular, scalable backbones tailored to this modality are missing.

### 1.3. Motivation and Study Outline

The essential motivation of this research is to address the given literature gaps. To fill these gaps, we have proposed a fully convolutional deep learning model termed TurkerNeXtV2. To create TurkerNeXtV2, we were inspired by PoolFormer [47]. In PoolFormer, they used a pooling function instead of multi-head self-attention. We were also inspired by ConvNeXt [48] to create our new-generation stem block. By proposing TurkerNeXtV2, we have introduced a fully convolutional deep learning model inspired by transformers, thereby addressing the first literature gap. Moreover, this contribution advances the development of new-generation deep learning models, filling the second literature gap. All team members are academics, and together they proposed this new-generation deep learning architecture. The design of TurkerNeXtV2 was divided into four main phases to ensure both clarity and functionality. The stem phase initializes the feature maps by using a ConvNeXt-inspired block, providing a strong yet lightweight foundation for later processing. The TNV2 phase serves as the core innovation, where pooling-based attention fused with an inverted bottleneck extracts informative features while keeping the model efficient. The hybrid downsampling phase addresses the routing problem by combining max pooling, average pooling, and grouped convolution, thereby preserving both global structure and local detail during resolution reduction. Finally, the output phase, based on global average pooling and a fully connected classifier, ensures scalability across different class settings and enables straightforward deployment. This four-phase division highlights the motivation to design a CNN that is modular, interpretable, and computationally efficient while still being capable of delivering transformer-level performance for biomedical image classification.

### 1.4. Innovations and Contributions

This study is innovative in both its architecture and its application. We introduce TNV2, a fully convolutional attention block that combines pooling-based attention with an inverted bottleneck. This design highlights focal pressure patterns without adding parameters, keeps the network lightweight, and yields interpretable saliency maps. We also propose a hybrid downsampling module that merges max pooling, average pooling, and grouped convolution to reduce routing bias and preserve detail at low cost. In addition, the stem is a modified ConvNeXt patchify stage with a light inverted bottleneck and fewer activations, which improves early features and training stability.

In this research, a lightweight (6.3–7.1 million parameters) version of the TurkerNeXtV2 has been recommended. We also provide a new plantar-pressure dataset and show that the model transfers well to blood-cell images, which indicates that it is practical and ready for clinical use.

Innovations:

- To our knowledge, this is the first dataset specifically tailored for CNN-based OA detection using plantar-pressure imaging.
- A new block was designed that combines max pooling and average pooling to form a pooling-based attention mechanism. This is fused with an inverted bottleneck, creating the TNV2 block as the main feature extractor. The multiple pooling functions improve stability, enhance feature selection, and reduce bias compared to earlier attention modules.
- Conventional downsampling often suffers from routing bias. To address this, a hybrid block was introduced that merges max pooling, average pooling, and patchified grouped convolution, followed by a 2 × 2 fusion layer. This design balances efficiency with richer spatial representation.
- The stem was simplified using a ConvNeXt-style structure, which improves early-stage feature learning and keeps the network modular.
- TurkerNeXtV2 differs from our previous backbones in three core ways. First, the main feature block (TNV2) replaces the MLP + transposed-convolution attention of Attention TurkerNeXt with a pooling-based attention (max + avg) fused to an inverted bottleneck, improving stability and efficiency. Second, the hybrid downsampling (max pool + avg pool + patchified grouped convolution) reduces routing bias versus the single 2 × 2 stride used previously. Third, the stem and head are simplified (ConvNeXt-style stem, GAP + FC head) to keep the model modular and scalable. Unlike our earlier TurkerNeXt pipeline, which added a hand-crafted deep-feature engineering stage with SVM, TurkerNeXtV2 is a compact, end-to-end CNN designed for plantar-pressure images.

Contributions:

- We collected and released a plantar-pressure image dataset, providing a valuable resource for biomedical engineering and clinical research.
- A lightweight CNN that integrates pooling-based attention, hybrid downsampling, and a ConvNeXt-inspired stem has been presented. This design advances the field of lightweight deep learning by demonstrating that compact models can deliver strong accuracy in both general and biomedical image classification.

## 2. Materials and Methods

### 2.1. Material

The primary objective of collecting a new plantar-pressure image dataset was to provide a reliable benchmark for the diagnosis of knee OA. Existing plantar-pressure datasets are scarce, often limited in size, and not specifically designed for OA detection, which restricts their utility for deep learning research. To address this gap, we constructed a dedicated dataset that captures the plantar-pressure distribution of both patients with clinically confirmed OA and healthy controls.

A total of 144 participants were enrolled in this study, including 54 patients with radiographically confirmed Kellgren–Lawrence (KL) Grade 4 knee osteoarthritis (OA) and 90 healthy controls with KL Grade 0 (no radiographic OA) (Table 2). All knee X-rays were independently reviewed by two experienced orthopedic surgeons, and only those fulfilling the KL Grade 4 criteria for OA or KL Grade 0 criteria for healthy controls were included. The OA group had a mean age of 65.27 ± 3.53 years (12 males, 22.2%; 42 females, 77.8%), while the control group had a mean age of 56.42 ± 6.21 years (39 males, 43.3%; 51 females, 56.7%). The mean height was 159.1 ± 4.77 cm in the OA group and 166.6 ± 7.24 cm in the control group. The mean body mass index (BMI) was 26.5 ± 5.10 kg/m^2^ in the OA group and 23.9 ± 2.87 kg/m^2^ in the control group. Controls were selected to be socio-demographically comparable to the patient group. For clarity, the Kellgren–Lawrence classification system used for radiographic grading is summarized below:

- Grade 0: No radiographic features of OA.
- Grade 1: Doubtful narrowing of joint space, possible osteophytic lipping.
- Grade 2: Definite osteophytes, possible narrowing of joint space.
- Grade 3: Multiple moderate osteophytes, definite joint-space narrowing, some sclerosis, possible bony deformity.
- Grade 4: Large osteophytes marked joint-space narrowing, severe subchondral sclerosis, and definite bony deformity.

All participants voluntarily joined the study, and written informed consent was obtained from each. The study was conducted in accordance with the principles of the Helsinki Declaration and approved by the relevant local ethics committee.

For inclusion in the patient group, participants must have had a confirmed diagnosis of stage 4 knee osteoarthritis and preserved walking function. Exclusions included individuals with a history of infected joint prostheses, limb amputations, Parkinson’s disease or severe depression causing balance issues during gait analysis, and those with active foot lesions (e.g., wounds/ulcers) potentially affecting plantar pressure measurements.

A dataset of plantar-pressure distributions and gait parameters was collected from both patient and control groups. The acquisition system was the Win-Track platform (MEDICAPTEURS Technology, Balma, France), a specialized device for the analysis of plantar pressure during barefoot walking. The system has a measurement surface of 1610 mm × 652 mm × 30 mm with a thickness of 9 mm, which provides sufficient area for recording complete steps.

The platform contains a dense array of pressure sensors that detect the vertical force applied by the foot at each point of contact. The sensors provide high spatial resolution across the entire plantar surface. The sampling frequency is up to 200 Hz, which allows accurate temporal analysis of foot loading and gait cycles. The device is capable of capturing both static stance and dynamic walking, with outputs that include pressure distribution, center of pressure trajectory, and temporal gait parameters.

The accuracy of measurement is ensured by the high density of sensors, the fast sampling rate, and the stable calibration process of the platform. These specifications make the Win-Track system suitable for the detection of small gait deviations that are often linked to musculoskeletal disorders such as knee osteoarthritis [49].

Participants walked barefoot across the Win-Track platform at a comfortable pace that they chose themselves. Each participant completed several trials to ensure that complete steps from both feet were recorded. The system detected foot contact with the sensors and converted each step into numerical values and visual pressure maps. Measurements included both static stance, when the participant stood on the platform for a few seconds, and dynamic walking, when the participant moved across the platform.

In addition to pressure maps, we also recorded videos of each trial. These videos were later processed, and pressure images were captured from the recorded videos to build the dataset. This ensured that the dataset contained both raw pressure recordings and standardized image representations for analysis.

The platform produced plantar-pressure images and gait curves for each step. The recorded data were transferred directly to a computer for analysis. Parameters such as foot contact duration, maximum pressure points, and center-of-pressure trajectory were calculated in each trial. The weight of each participant was measured before the trials and entered into the software, so that the plantar-pressure values could be normalized and compared fairly between groups.

This procedure generated both static and dynamic measurements, together with synchronized video-derived images, which allowed a meaningful evaluation of gait patterns. Differences between patients with knee osteoarthritis and healthy controls were assessed based on pressure distribution, balance, and spatiotemporal gait characteristics.

The collected dataset contains pressure images and the distribution of these images have been given in Table 3.

### 2.2. TurkerNeXtV2

The primary objective of this research is to design and present TurkerNeXtV2, a new lightweight CNN that integrates innovative architectural blocks to achieve high classification performance in biomedical image analysis. The model is divided into four main phases: a ConvNeXt-inspired stem, the proposed TNV2 block, a hybrid downsampling block, and an output block. The key aim is to create a CNN that delivers transformer-level effectiveness while maintaining the simplicity, efficiency, and deployability of convolutional models. To demonstrate its capability, TurkerNeXtV2 is applied to knee osteoarthritis detection from plantar-pressure images and further tested on a blood-cell image dataset for cross-domain validation. In addition to accuracy, the objectives include evaluating computational efficiency in terms of parameters, FLOPs, and inference speed, and providing explainable results through Grad-CAM to support practical use in clinical environments.

To achieve high classification performance, we drew inspiration from transformers and ConvNeXt; thus, a modified ConvNeXt block is employed as the stem.

The main innovation in the TNV2 block is the fusion of a pooling-based attention mechanism with an inverted bottleneck:

Pooling-based attention replaces heavy self-attention:

- It uses parallel max-pooling and average-pooling to capture both focal pressure deviations and global context.
- Their element-wise product, gated by a sigmoid, suppresses noise and highlights clinically relevant regions.

Inverted bottleneck (like in transformers) is then stacked on this attention:

- It expands–compresses feature via depth-concatenation and grouped convolutions, keeping the block lightweight.

Together, these two components create a fully convolutional, low-cost, interpretable attention unit that serves as the primary feature-map generator in TurkerNeXtV2.

Downsampling is critical for CNNs, and a routing problem often arises. Many researchers therefore prefer convolution-based downsampling over pooling-based approaches. In TurkerNeXtV2, we combine max pooling, average pooling, and grouped convolution to create a hybrid downsampling block.

**Figure 1:**
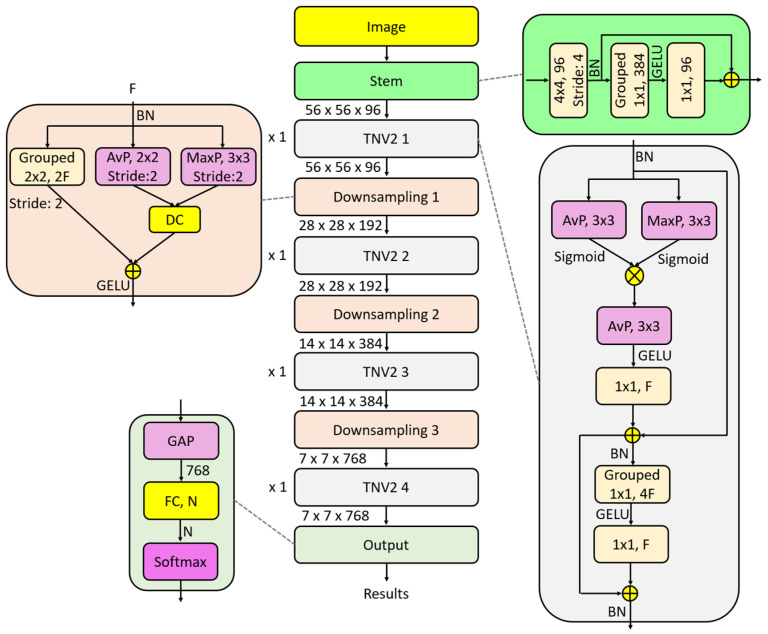
The graphical overview of the TurkerNeXtV2. * BN: Batch Normalization; AvP: Average Pooling; MaxP: Max Pooling; DC: Depth Concatenation; GELU: Gaussian Error Linear Unit; GAP: Global Average Pooling; FC: Fully Connected Layer; N: Number of classes; Stem: initial feature-extraction block; TNV2: TurkerNeXtV2 block; Downsampling: stage with stride 2 fusion; Grouped k × k, c: grouped convolution (kernel k × k, output c channels); 1 × 1, c: pointwise convolution with c channels; 3 × 3: 3 × 3 kernel (conv or pool as labeled); Stride: step size of pooling/conv; Sigmoid: gating activation; F: channel width at the current stage; 2F/5F: 2×/5 × F output channels; “×1” near a block: number of repeats; 56 × 56 × 96 etc.: feature-map size (H × W × C); Output/Softmax: classifier head producing class probabilities.

Finally, the output block uses global average pooling for flattening, a fully connected layer to set the number of classes, and a softmax layer to generate classification results. A graphical overview of TurkerNeXtV2 is provided in Figure 1.

The network has four phases: a ConvNeXt-inspired stem, the TNV2 block, a hybrid downsampling block, and an output head. The input size is 224 × 224 × 3. Feature maps are shown from left to right. The stem converts the image to a 56 × 56 × 96 tensor by a 4 × 4 convolution with stride 4. TNV2 applies pooling-based attention and an inverted bottleneck. Hybrid downsampling combines max pooling, average pooling, and grouped convolution. The head uses global average pooling and a fully connected layer for classification. Shapes are indicative.

The pseudocode of the recommended TurkerNeXtV2 (lightweight version; see Figure 1) is presented in Algorithm 1.
**Algorithm 1.** Pseudocode of the TurkerNeXtV2**Input:** Image with a size of 224 × 224 × 3, **Output:** Classification results.01: Apply the ConvNeXt-modified stem block to generate the first tensor of size 56 × 56 × 96. 02: Pass the first tensor through the first TNV2 block to create the second tensor of size 56 × 56 × 96. 03: Use the first hybrid downsampling block to create a new tensor of size 28 × 28 × 192. 04: Apply the second TNV2 block to the downsampled tensor to create the third tensor of size 28 × 28 × 192. 05: Deploy the second hybrid downsampling block to create a new tensor of size 14 × 14 × 384. 06: Apply the third TNV2 block to generate a tensor of size 14 × 14 × 384. 07: Apply the third hybrid downsampling block to produce the final downsampled tensor of size 7 × 7 × 768. 08: Generate the last tensor by applying the fourth (final) TNV2 block; its size remains 7 × 7 × 768. 09: Perform GAP to obtain 768 features. 10: Use an FC layer to set the number of classes. 11: Apply the softmax function to produce classification outputs.

Furthermore, we have given the transitions of the TurkerNeXtV2 in Table 4.

Table 4 illustrates the recommended model’s repetition counts and filter numbers provided below.

(1) $$TurkerNeXtV2=F:\left\{96,192,384,768\right\},\;R:\left\{1,1,1,1\right\}\;$$

Herein, F: number of filters and R: number of the repetitions. Also, Table 4 demonstrated that the introduced TurkerNeXtV2 is a lightweight CNN architecture. The details of the utilized phases in the presented TurkerNeXtV2 are explained below.

#### 2.2.1. Stem

We adopt the ConvNeXt patchify idea in the stem but tailor it for a lightweight start and stable training. First, we keep the ConvNeXt attribute of a 4 × 4 convolution with stride 4 and 96 output channels, which converts the input (224 × 224 × 3) into a compact 56 × 56 × 96 tensor with very low cost. Second, we insert a small inverted bottleneck on top of this tensor: a 1 × 1 grouped convolution expands the channels to 4F (384), a single GELU provides the only nonlinearity, and a 1×1 projection brings the channels back to 96. Third, we use batch normalization and a residual addition from the patchify path to stabilize optimization.

This modification reduces the number of activations (only one GELU in the stem), preserves information after aggressive downsampling, and enriches early features without adding many parameters. In short, the stem keeps the ConvNeXt spirit (patchify + simple blocks) but adds a light inverted bottleneck and residual path to improve robustness and efficiency for TurkerNeXtV2. Equation (2) formalizes this design.

(2) $${T_{1}=C}_{96}^{1\times 1}\left(GELU\left(GC_{384}^{1\times 1}\left(BN\left(C_{96,St=4}^{4\times 4}\left(Im\right)\right)\right)\right)\right)+BN\left(C_{96,St=4}^{4\times 4}\left(Im\right)\right)\;\;$$

where T: tensor and $T_{1}$: the first tensor; C(.): convolution function; Im: input image with a size of 224 × 224 × 3; St: stride value; BN(.): batch normalization; GC(.): grouped convolution function and GELU(.): GELU activation function. By deploying this formula (see Equation (1)), a tensor with a size of 56 × 56 × 96.

#### 2.2.2. TurkerNeXtV2 Block

The TNV2 block is the main feature generator of TurkerNeXtV2 and represents a fully convolutional adaptation of transformer-inspired designs. It combines a pooling-based attention mechanism with an inverted bottleneck to create an efficient and interpretable unit. Unlike conventional self-attention, which is computationally expensive, pooling-based attention leverages parallel average pooling and max pooling to capture both global context and local salient patterns in the input feature map. The fusion of these pooling operations, regulated by a sigmoid gate, suppresses noise and highlights diagnostically relevant regions [47,50]. On top of this attention module, an inverted bottleneck structure—widely used in lightweight CNNs such as MobileNetV2 [51] and later adopted in ConvNeXt [48]—is applied to expand and compress features through depthwise or grouped convolutions and we used fewer normalization and activations. This design enhances feature richness while keeping parameter count. Together, pooling-based attention and the inverted bottleneck form the TNV2 block, a compact yet powerful convolutional unit that improves stability, interpretability, and efficiency in CNN architectures.

In this block, we aim to propose a fully convolutional version of PoolFormer. Therefore, we present a pooling-based attention mechanism and add an inverted-bottleneck block to it. By using pooling-based attention, the model can focus on regions of interest (ROIs), while the inverted bottleneck extracts meaningful features from each ROI. Thus, we generate informative features with the proposed TNV2 block. The mathematical definition of this block is given below.

(3) $$T_{n}^{Att_{1}}=S\left(P_{3\times 3}^{A}\left(BN\left(T_{n-1}\right)\right)\right)*S\left(P_{3\times 3}^{M}\left(BN\left(T_{n-1}\right)\right)\right)$$

(4) $$T_{n}^{Att_{2}}=DC\left(T_{n}^{Att_{1}},BN\left(T_{n-1}\right)\right)$$

(5) $$T_{n}^{Att}=C_{F}^{1\times 1}\left(GELU\left(P_{3\times 3}^{A}\left(T_{n}^{Att_{2}}\right)\right)\right)+BN\left(T_{n-1}\right)\;$$

Above, we have defined pooling-based attention block. In this block, matrix multiplication, depth concatenation and residual block have been utilized together, where S(.): sigmoid function; $P^{A}(.)$: average pooling; $P^{M}(.)$: maximum pooling; and $T^{Att}(.)$: attention tensor.

The pooling-based attention block was selected because it provides a simple and effective mechanism to increase feature representation without increasing the number of parameters. Unlike conventional self-attention, which requires heavy matrix multiplications and additional trainable weights, pooling operations act as fixed filters that capture both global and local information directly from the feature maps. In TurkerNeXtV2, average pooling captures the global pressure distribution, while max pooling emphasizes local deviations or focal abnormalities. Their element-wise fusion highlights clinically relevant regions and suppresses noise.

This design choice ensures that the model remains lightweight and computationally efficient, making it more suitable for medical imaging applications where datasets are modest and deployment often requires resource-constrained environments such as mobile or point-of-care device.

An inverted bottleneck block has been added to this attention block like transformers.

(6) $$T_{n}=C_{F}^{1\times 1}\left(GELU\left(GC_{4F}^{1\times 1}\left(BN\left(T_{n}^{Att}\right)\right)\right)\right)+T_{n}^{Att}$$

Herein, we have used inverted bottleneck. The inverted bottleneck was originally introduced in MobileNetV2 to improve efficiency by expanding channels before depthwise convolution and then compressing them back to a smaller dimension. This strategy allows the network to process richer features in the expanded space without a large increase in parameters, and then reduce dimensionality to maintain a lightweight structure.

A similar concept has also been adopted in transformer-inspired architectures, where feed-forward networks follow the same expand–compress principle. In transformers, the hidden dimension of the feed-forward layer is expanded to a much larger size, nonlinear transformations are applied, and then the representation is projected back to the original dimension. This expand–compress cycle increases the model’s representational capacity while controlling computational cost.

In TurkerNeXtV2, the inverted bottleneck plays the same role: it enriches the features extracted by the pooling-based attention while keeping the architecture compact.

The equations above define the proposed TNV2 block.

#### 2.2.3. Hybrid Downsampling

To avoid the routing problem, we use two pooling operations and a grouped convolution. To prevent peak- or average-value routing, we combine average pooling and maximum pooling, and we apply grouped convolution to obtain the downsampled tensor. The mathematical definition of the hybrid downsampling block is given below.

(7) $$T_{D}=GELU\left(DC\left(P_{2\times 2,St=2}^{A}\left(T_{n-1}\right),P_{2\times 2,St=2}^{M}\left(T_{n-1}\right)\right)+GC_{2F,St=2}^{2\times 2}\left(T_{n-1}\right)\right)$$

Herein, $T_{D}$: downsampled tensor.

#### 2.2.4. Output

To generate the classification results, a straightforward output block is used to demonstrate the classification ability of the extracted features. In this phase, GAP flattens the final tensor, an FC layer sets the number of classes, and the softmax function produces the classification outputs. The mathematical definition of this block is given below.

(8) $$out=SM\left(FC\left(\left(GAP\left(T_{last}\right)\right),N_{C}\right)\right)$$

where out: classification outcome; SM(.): softmax function; FC(.): fully connected; $N_{C}$: number of classes; GAP(.): global average pooling and $T_{last}$: the generated last tensor with a size of 7 × 7 × 768.

## 3. Experimental Results

### 3.1. Experimental Setup

**Figure 2:**
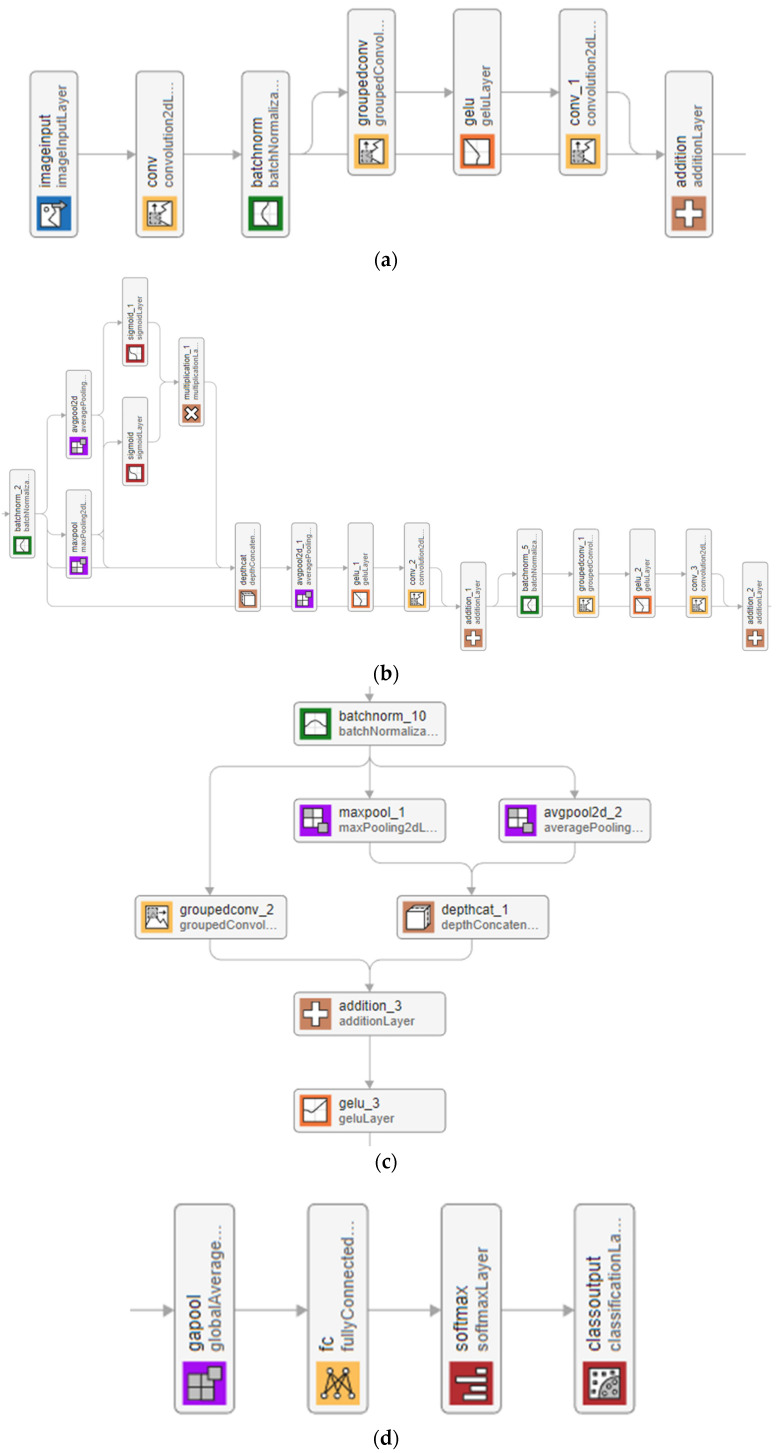
Blocks used in TurkerNeXtV2 implemented in MATLAB DND. (**a**) Stem block; (**b**) TNV2 block; (**c**) Hybrid Downsampling; (**d**) Output.

In this research, the classification results of the proposed TurkerNeXtV2 are presented. TurkerNeXtV2 was designed on a simply configured personal computer (PC) equipped with 128 GB of main memory (Corsair, Fremont, CA, USA), a 1.82 TB storage (Samsung, Suwon, Republic of Korea), an Intel i9-14900KF processor (Intel, Santa Clara, CA, USA), a GeForce RTX 5080 graphics processing unit (GPU) with 16 GB of memory (NVIDIA, Santa Clara, CA, USA), and the Windows 11 operating system. The programming environment used is MATLAB 2023a, specifically the MATLAB Deep Network Designer (DND). Within DND, we employed 97 layers and 127 connections. This design is shown in Figure 2.

In Figure 2a, the stem stage begins the feature extraction process. It uses convolution, batch normalization, grouped convolution, GELU activation, and a residual addition. The aim is to produce strong initial feature maps while keeping the number of parameters small.

In Figure 2b, the main block is defined and it is TNV2 block. This block combines average pooling and max pooling in parallel to form pooling-based attention. This operation highlights both global context and local pressure changes without adding extra parameters. A sigmoid gate fuses the two signals, reducing noise and focusing on clinically relevant regions. On top of this, an inverted bottleneck expands and compresses features through grouped convolutions, producing rich representations at low cost.

Hybrid downsampling stage (see Figure 2c) reduces the spatial size of the feature maps. It merges three operations: max pooling, average pooling, and grouped convolution. Their outputs are fused through concatenation and a 2 × 2 convolution. This approach preserves detailed information while lowering resolution, avoiding the routing bias that occurs when only a single pooling operator is used.

In Figure 2d, the output stage is defined. This stage uses global average pooling, a fully connected layer, a softmax function, and a classification layer. This design transforms the feature maps into class probabilities with very few parameters. It also improves generalization by reducing the risk of overfitting.

The chosen blocks meet three key criteria. First, they keep the model lightweight by reducing parameters. Second, they provide stable optimization through batch normalization, residual connections, and GELU activation. Third, they improve interpretability by using pooling attention, which functions as a built-in saliency filter. All components are available in MATLAB DND, which ensures reproducibility.

### 3.2. Training Configuration

TurkerNeXtV2 was trained in MATLAB 2023a using the default training settings of DND. The use of these parameters follows the standard defaults of MATLAB DND, which are commonly applied in CNN studies and provide a stable starting point. SGDM with momentum is a standard optimizer for CNNs. A batch size of 128 provides a good balance between GPU memory and training speed. The learning rate of 0.01 is widely accepted in the literature as a reliable choice for stable convergence. The default weight decay of 1 × 10^−4^ also reduces the risk of overfitting without adding extra complexity.

To train the TurkerNeXtV2 CNN, we used the following parameters:

Solver: Stochastic Gradient Descent with Momentum (SGDM),
Standard MATLAB default; widely used optimizer for CNNs; stable convergence.
Number of Epochs: 30,
MATLAB default; sufficient for convergence on the datasets used.
Batch Size: 128,
Balanced choice for GPU memory and training speed; MATLAB default setting.
Initial Learning Rate: 0.01,
Common default in CNN training; reliable for stable convergence in many vision tasks.
L2 Regularization: 1 × 10^−4^,
Default weight decay; reduces risk of overfitting without extra complexity.
Training–Validation Split: 80:20, randomized.

To choose these hyperparameters, we did not use hyperparameter tuning method (e.g., Bayesian optimization or multi-fidelity bandits). These are MATLAB’s default settings.

### 3.3. Pretraining on Stable ImageNet-1k

Stable ImageNet-1k is a synthetic version of the ImageNet dataset that was generated to provide a standardized and reproducible resource for model training. The archive is organized into 1000 folders, each corresponding to one class. Every folder contains 100 generated images, giving a total of 100,000 images.

The Stable ImageNet-1k dataset is important for TurkerNeXtV2 because it provides a large-scale but balanced training set where every class has the same number of images. Unlike the original ImageNet, which has uneven class distributions and potential noise in labeling, Stable ImageNet-1k offers cleaner, class-balanced, and reproducible data. Pretraining on this dataset allowed TurkerNeXtV2 to learn general visual features across 1000 diverse categories, which improved transfer learning performance when fine-tuned on smaller biomedical datasets such as plantar-pressure images and blood-cell images.

With these settings, we first trained on Stable ImageNet-1k to obtain a pretrained CNN, achieving the validation results shown in Table 5.

Table 5 demonstrates that the proposed TurkerNeXtV2 achieved a final validation accuracy of 87.77% on Stable ImageNet-1k. This was computed on a held-out validation split as the proportion of images for which the predicted class (softmax argmax) matched the ground-truth label. The corresponding losses and accuracies were training loss = 0.0331, training accuracy = 100%, validation loss = 0.4647, and validation accuracy = 87.77% (Table 5). This result indicates that the backbone learns generalizable features beyond the training data; the expected train–validation gap reflects task difficulty (1000 classes) rather than overfitting, since validation loss remains moderate and accuracy is high without any hyperparameter tuning. We have used this pretrained dataset to obtain high classification performances on the curated dataset.

### 3.4. Training on Osteoarthritis Dataset

**Figure 3:**
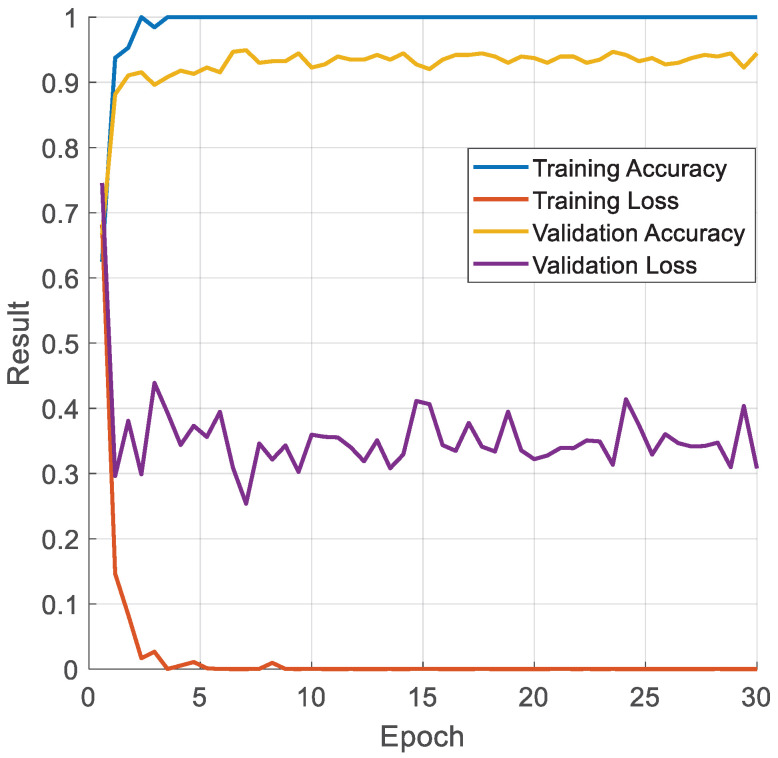
Training and validation curves on the osteoarthritis pressure-image dataset.

In the second step, we fine-tuned the pretrained TurkerNeXtV2 on the collected pressure-image dataset; the resulting metrics are presented in Figure 3.

TurkerNeXtV2 was trained for 30 epochs using SGDM, batch size 128, initial learning rate 0.01, L2 = 1 × 10^−4^, and an 80:20 training–validation split. The model was pretrained on Stable ImageNet-1k and then fine-tuned here. Curves show rapid convergence and a small generalization gap. Final values are training accuracy 100%, validation accuracy 93.72%, training loss 1.52 × 10^−6^, and validation loss 0.3168.

Training curves (Figure 3) showcase rapid convergence. Training accuracy reaches ≈1.00 by epoch 3. Validation accuracy stabilizes at 0.93–0.95, and the generalization gap remains small (≈0.04–0.06). Validation loss fluctuates around 0.35 without an upward trend. These patterns do not indicate underfitting or overfitting and confirm that model capacity is appropriate for this dataset.

The TurkerNeXtV2 was trained with a standard optimizer. We also used Stable ImageNet-1k pretraining, checkpoint selection by validation accuracy. These choices reduce variance and support stable generalization on a modest dataset.

According to Figure 3, the computed training and validation results are presented in Table 6.

Table 6 shows that the proposed TurkerNeXtV2 achieved 93.72% validation accuracy.

### 3.5. Test Evaluation

**Figure 4:**
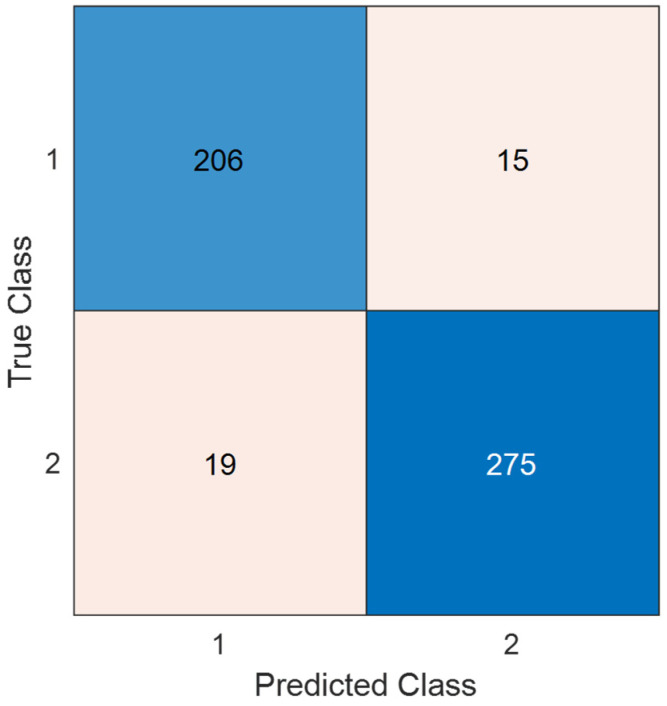
Test confusion matrix for the osteoarthritis pressure-image dataset. Herein, 1: Osteoarthritis, 2: Control.

Table 5 reports the performance of TurkerNeXtV2 during pretraining on the large-scale Stable ImageNet-1k dataset, where the model reached a validation accuracy of 87.77%. This stage highlights the general representational capacity of the backbone on a standard benchmark. Table 6 then presents the results after adapting the pretrained model to our osteoarthritis pressure-image dataset, achieving a validation accuracy of 93.72% with minimal loss. This confirms effective transfer to the target domain. Finally, Figure 4 shows the independent test evaluation, with the confusion matrix summarizing classification outcomes. In summary, Table 5 reflects pretraining, Table 6 reflects domain adaptation, and Figure 4 shows final test performance.

Rows denote true class and columns denote predicted class. Class 1 is Osteoarthritis and Class 2 is Control. Counts are from the independent test set (*n* = 515; 221 OA and 294 Control). The matrix is the source for the test metrics in Table 7, including accuracy, precision, recall (sensitivity), specificity, F1-score, and balanced accuracy.

Per Figure 4, the computed test classification performances are listed in Table 7.

The overall test classification metrics are tabulated in Table 7, which clearly shows that TurkerNeXtV2 achieved over 90% on every performance metric. Moreover, it reached 93.21% sensitivity, 93.54% specificity, and 93.37% geometric mean (support class is Osteoarthritis). Moreover, balanced accuracy has been computed as 93.38%.

On the independent test set (*n* = 515), the model achieved 93.4% accuracy (95% CI 91.3–95.2%). A one-sided binomial test confirmed that the observed accuracy is significantly higher than chance (50%) and also higher than a strong baseline of 85% (*p* < 0.001). This highlights that the model’s performance is statistically robust and not due to random variation.

The test confusion matrix (see Figure 4) explains how the model behaves on the independent test set (data not used for training or validation). It showcases correct decisions and the two error types—false negatives (missed disease) and false positives (healthy mislabeled as disease)—for each class. In the osteoarthritis task, the matrix reports 206 true OA correctly detected, 15 OA missed, 275 controls correctly identified, and 19 controls mislabeled as OA. These counts are the basis for the test metrics in Table 7 (precision, recall/sensitivity, specificity, F1-score, balanced accuracy) and therefore give a transparent view of clinical risk.

### 3.6. Computational Efficiency

The second criterion is computational efficiency. We measured the inference time of TurkerNeXtV2 and compared it with a range of established CNN and transformer baselines under the same RTX 5080 hardware and MATLAB implementation. Table 8 lists the average prediction time, throughput (images/s), and relative performance compared to TurkerNeXtV2. This evaluation highlights the runtime characteristics of the proposed model in relation to both lightweight CNNs and heavier state-of-the-art architectures.

As shown in Table 8, TurkerNeXtV2 processes 128.8 images/s, demonstrating efficiency comparable to GoogLeNet (141.9 images/s) and outperforming MobileNetV2 (105.6 images/s), InceptionV3 (72.3 images/s), DenseNet201 (23.7 images/s), and transformer-based ViT (46.1 images/s). With an average inference time of 0.0078 s, the proposed model offers a favorable balance between speed and accuracy, ensuring practical applicability for real-time and clinical environments.

### 3.7. Explainability

**Figure 5:**
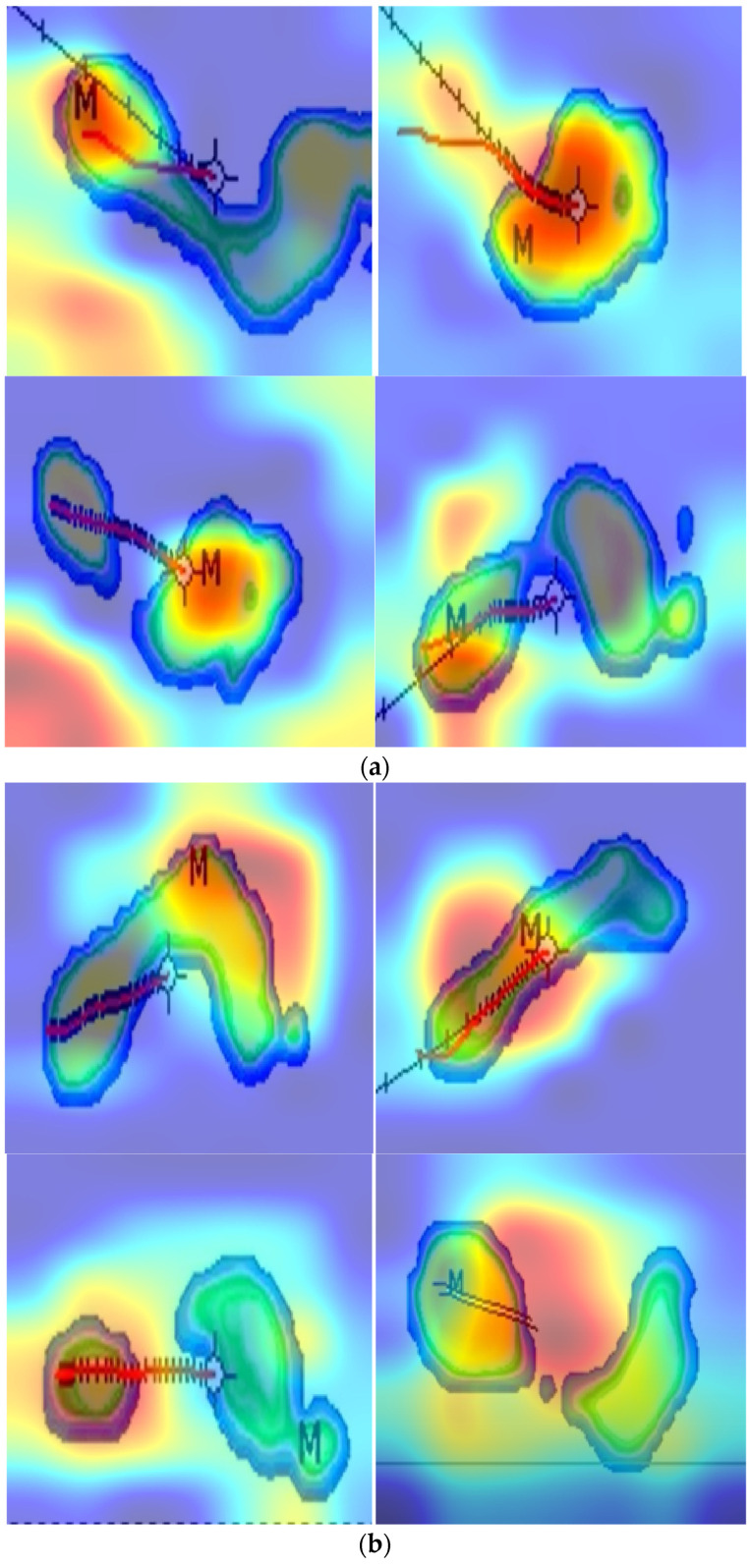
Grad-CAM heatmap images of some sample images. (**a**) Osteoarthritis; (**b**) Control. Red/yellow indicates higher model contribution and blue/green lower contribution. Labels mark the high-intensity core, the strongest pressure area (M from the Win-Track software), the transition zone, the foot outline, and the center-of-pressure (COP) trajectory. Maps were computed from the last convolutional layer. The visual focus aligns with biomechanically meaningful regions and supports the role of pooling-based attention in TNV2.

The third evaluation criterion is explainability. To provide interpretable results, Gradient-weighted Class Activation Mapping (Grad-CAM) was applied, and the resulting heatmaps for sample images are shown in Figure 5.

Figure 5 illustrates Grad-CAM heatmaps over Win-Track plantar-pressure images. In osteoarthritis (panel a), a compact red/yellow core marks the TurkerNeXtV2 attention peak on the strongest pressure zone (M mark from Win-Track). The center-of-pressure (COP) trajectory passes through this core, confirming that the network emphasizes biomechanically meaningful regions. In controls (panel b), activations are broader and lower in intensity (green/blue), consistent with a more even load distribution. Labels indicate “High-intensity core (TNV2 attention peak),” “Strongest pressure area (M),” and “COP trajectory.” An inset schematic explains the mechanism: max pooling isolates local hotspots, average pooling captures global distribution, and gated fusion forms the final attention map. The figure demonstrates that the model amplifies clinically relevant hotspots in osteoarthritis and suppresses background in controls.

In this study, focal pressure refers to a local peak relative to its neighborhood and to the subject’s normalized load. No fixed absolute threshold is required. Win-Track software normalizes pressures by body weight, so the model attends to relative peaks. The TNV2 block identifies these peaks through pooling-based attention. Max pooling highlights concentrated loads, average pooling summarizes the overall map, and the gated fusion highlights regions that stand out in a single step.

Deviations from focal pressure alter the contrast of the map rather than individual values. Osteoarthritis cases show compact, high-intensity peaks at heel or forefoot, producing strong attention responses and higher class confidence, visible as red/yellow clusters in panel a. Controls show broad, low-intensity loads, yielding diffuse responses (panel b) consistent with balanced gait. Off-loading or displaced peaks reduce the attention response and lower confidence, but the average-pool path preserves context and prevents instability. Spurious peaks from partial steps or sensor noise may cause false saliency; weight normalization, batch normalization, and independent test evaluation limit this risk. Pooling-based attention aggregates features with max pooling and average pooling and then applies a gate, so only activations that are high both locally and with respect to the global load pass with strong weight. This acts as an intrinsic denoising step and suppresses isolated, spurious peaks—an effect consistent with CBAM [52], which uses max + average pooling to refine features with negligible overhead and reports better localization and recognition performance.

As a complementary line of evidence, PoolFormer shows that replacing heavy token mixers with simple pooling can still yield competitive accuracy at lower parameter and compute cost, supporting the idea that pooling-based mixing [47] can produce clean, discriminative feature maps.

Grad-CAM localizes class-relevant regions by using gradients at the last convolutional layer; when intermediate features are cleaner and more concentrated, the resulting attribution maps tend to be tighter and better aligned with true hot spots.

Thus, Figure 5 visualizes how TNV2 reacts to focal-pressure patterns: it amplifies clinically meaningful hotspots and suppresses irrelevant regions, providing transparent, step-level evidence for the test results reported in the paper.

The pooling-based attention sharpens the Grad-CAM heatmaps and suppresses false-positive highlights. The smooth transitions along cartilage boundaries and the absence of noisy artefacts demonstrate that the mechanism balances sensitivity to local abnormalities with robustness to imaging noise. These observations confirm that the lightweight pooling strategy embedded in TurkerNeXtV2 delivers interpretable focus maps that are both clinically coherent and computationally economical.

## 4. Discussion

**Figure 6:**
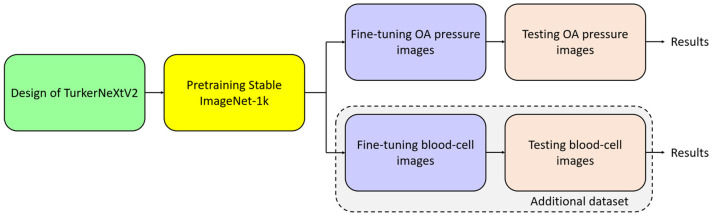
Study pipeline.

We propose TurkerNeXtV2, a lightweight CNN that joins a ConvNeXt-inspired stem, the TNV2 block (pooling-based attention + inverted bottleneck), a hybrid downsampling layer, and a simple output head. The aim is to combine the efficiency of convolutions with transformer-like effectiveness while remaining interpretable. The overall study pipeline is showcased in Figure 6.

Convolutions reuse the same small kernels across the image, which gives strong feature extraction with weight sharing and good memory locality. This usually leads to lower latency and higher throughput on GPUs and embedded devices compared with heavier attention blocks. In practice, an efficient convolutional model should be small in size, fast at inference, and stable to train, without sacrificing accuracy.

TurkerNeXtV2 is built to meet this goal. The stem uses a 4 × 4 convolution with stride 4 to “patchify” the input and form a 56 × 56 × 96 tensor at very low cost. The TNV2 block uses pooling-based attention that adds no trainable parameters, then applies an inverted bottleneck with grouped and 1 × 1 convolutions to enrich features while keeping compute low. The hybrid downsampling combines max pooling, average pooling, and grouped convolution to reduce resolution without losing salient information. The head uses global average pooling and a single fully connected layer, which avoids heavy dense layers.

The experimental and visual results demonstrate that TurkerNeXtV2 merges convolutional efficiency with transformer-inspired attention while remaining lightweight and highly interpretable. The model achieved 93.40% test accuracy on the collected osteoarthritis pressure-image dataset, alongside balanced precision and recall (~93%), confirming its suitability for clinical screening tasks. Comparable performance on the Stable ImageNet-1k (1000-class) benchmark—87.77% validation accuracy after 30 epochs—further validates the architecture’s generalization capacity beyond the medical domain.

Central to this outcome is the pooling-based attention mechanism embedded in the TNV2 block. By combining max- and average-pooled feature maps through a sigmoid-gated, element-wise product, the network highlights regions exhibiting both focal pressure deviations (max pooling) and broader load imbalances (average pooling).

Explainability and efficiency are evaluated to support deployment. Grad-CAM heat maps indicate that the network focuses on focal loading zones in osteoarthritis and shows diffuse, low-intensity patterns in controls, which is consistent with the design of the pooling-based attention in TNV2 (see Figure 5). Inference time and throughput are measured under a common hardware and software setting, showing that TurkerNeXtV2 is fast enough for real-time analysis and compares with widely used baselines (Table 7).

The hybrid downsampling block concatenates max-pooled, average-pooled, and grouped-convolutional pathways, mitigates the “routing” problem often encountered when aggressive pooling discards fine-grained cues. This design preserves salient edges and global context while halving spatial resolution, maintaining discriminative power without increasing parameter count; the entire network remains ~7.1 M trainable weights for the 1000-class ImageNet-1k variant and ~6.3 M for the 10-class osteoarthritis task. Training on a single RTX 5080 GPU (it has 16 GB memory) converged within 30 epochs. This situation confirms the model’s accessibility to modestly equipped laboratories.

OA changes gait and plantar load in two ways: it creates local hotspots of pressure (focal loading) and global imbalance across the foot. The TNV2 block is built for this pattern. Max pooling highlights the local hotspots; average pooling captures the global distribution; their gated fusion focuses the model on relative peaks without adding new parameters. This reduces overfitting on a modest, single-center dataset and keeps training stable (Figure 3; Table 6). The inverted bottleneck then expands and compresses channels to enrich features at low cost, so the network can detect subtle shape and trajectory changes (e.g., around heel and forefoot) but stay lightweight. The result is high test accuracy on OA (Figure 4, Table 7) and clear heat maps that align with focal loading zones and the COP path (Figure 5). Finally, the design is fast (Table 8), which suits gait labs and clinics that already use pressure plates.

The proposed TurkerNeXt’s choices lowered validation accuracy on Stable ImageNet-1k (Attention TurkerNeXt 82.36%, TurkerNeXt 83.55%) compared with TurkerNeXtV2 (87.77%) under the same 30-epoch recipe, and they did not provide the same, clean saliency seen in Figure 5. They also used single-operator downsampling, which increases routing bias and can drop fine pressure details; TNV2’s hybrid downsampling preserves them.

Pooling attention is parameter-free and behaves like a built-in saliency filter, so it is robust to noise and device variation and easier to generalize. The inverted bottleneck adds expressive power at low compute. Together they deliver accuracy, speed, and interpretability: 93.40% test accuracy on OA with balanced precision/recall (Table 7), near-99% validation on blood cells (see Table 9) with strong test metrics, and real-time throughput (see Table 10 for blood-cell images test results). These gains come from the architecture, not hyperparameter search (MATLAB defaults).

TNV2 relies on contrast in the pressure map; extremely flat or noisy steps can weaken attention. It does not model very long-range interactions as fully as self-attention. Future work (TurkerNeXtV3) will explore hybrid pooling + MHSA and multi-centre datasets to address these points.

### 4.1. Test Additional Dataset

In this research, we collected a new dataset; therefore, we cannot provide comparative results for the collected pressure-image dataset. Consequently, we tested the proposed TurkerNeXtV2 on a publicly available blood-cell image dataset that contains eight classes, each representing a distinct blood cell [14,15]. The details of the utilized blood cell image dataset are given below.

The blood cell dataset contains a total of 17,092 RGB images distributed into eight categories. Each image shows a single blood cell type on a background of normal erythrocytes, and all images were resized to 360 × 363 pixels for consistency. The dataset was divided into 12,831 training samples (75.1%) and 4261 testing samples (24.9%), preserving the original class proportions.

For the basophil class, there are 914 training and 304 testing images, giving a total of 1218 samples. The eosinophil class includes 2340 training and 777 testing images, for a total of 3117 samples. The erythroblast class has 1164 training and 387 testing images, resulting in 1551 samples.

In the case of immature granulocytes, there are 2172 training and 723 testing images, with a total of 2895 samples. The lymphocyte class is smaller, with 912 training and 302 testing images, adding up to 1214 samples. The monocyte class includes 1068 training and 352 testing images, with a total of 1420 samples.

The largest class, neutrophils, is represented by 2497 training and 832 testing images, giving a total of 3329 samples. Finally, the platelet class consists of 1764 training and 584 testing images, with a total of 2348 samples.

This balanced split ensures that both frequent and less frequent blood cell types are sufficiently represented in the training and testing phases to support reliable evaluation of model performance across all categories.

**Figure 7:**
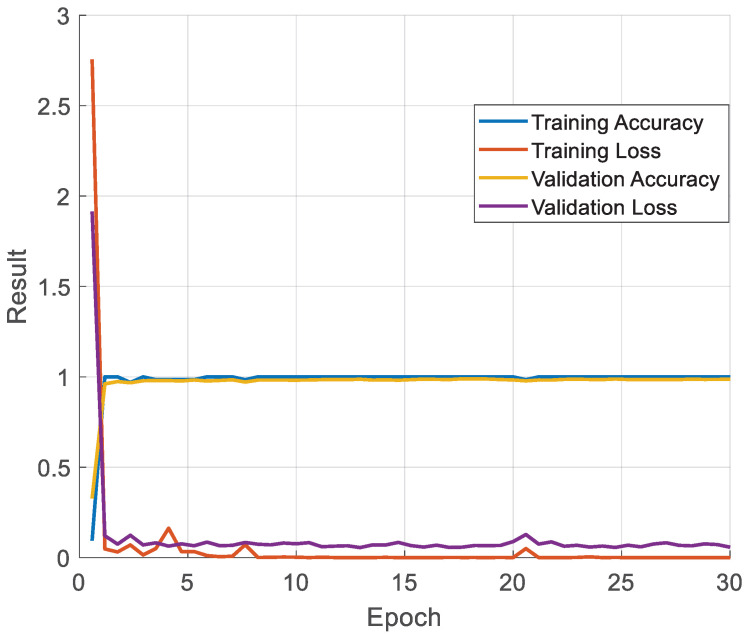
Training and validation curves on the blood-cell image dataset.

The training and validation curves for TurkerNeXtV2 on this dataset are shown in Figure 7.

Figure 7 showcases rapid convergence within the first few epochs. Training accuracy reaches 100% by approximately the third epoch and remains stable. Validation accuracy rises quickly and stabilizes around 98–99%, with only small fluctuations. Both training and validation losses decrease to very low values and remain stable throughout the 30 epochs. This pattern shows that the model does not suffer from underfitting (low accuracy in both training and validation) or overfitting (large and widening gap between training and validation). Instead, the curves confirm that TurkerNeXtV2 has sufficient capacity for this dataset and generalizes well.

According to Figure 7, the validation and training results of TurkerNeXtV2 on the blood-cell image dataset are presented in Table 9.

These results (Table 9) show that the design of TurkerNeXtV2, which uses pooling-based attention, the inverted bottleneck, and hybrid downsampling, achieves very high accuracy while keeping the model lightweight. The combination of high validation accuracy with low loss means that the model not only classifies correctly but also produces confident predictions, which is especially important in medical applications.

The blood cell dataset contains several classes that look very similar, which makes classification difficult for standard CNNs. TurkerNeXtV2 reached nearly 99% validation accuracy, showing that it can detect subtle differences in cell appearance. The stable training and validation curves confirm that the model is reliable, robust, and suitable for biomedical image classification.

**Figure 8:**
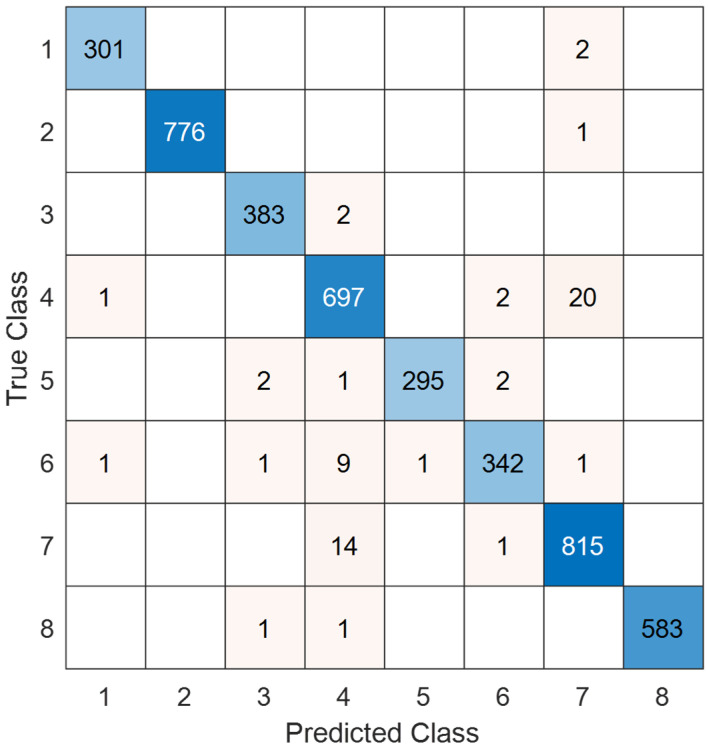
Test confusion matrix for the blood-cell image dataset. Rows denote true class and columns denote predicted class across eight cell categories. Cells show the number of test images assigned to each class. The matrix reveals where confusion occurs among visually similar categories and supports the per-class precision and recall analysis.

Using the pretrained TurkerNeXtV2 on the blood-cell image dataset, we computed the test results and present the confusion matrix in Figure 8.

By utilizing the confusion matrix showcased in Figure 8, the overall test classification performances are tabulated in Table 10.

In Table 10, classification metrics are computed on the independent test split for the eight classes.

**Figure 9:**
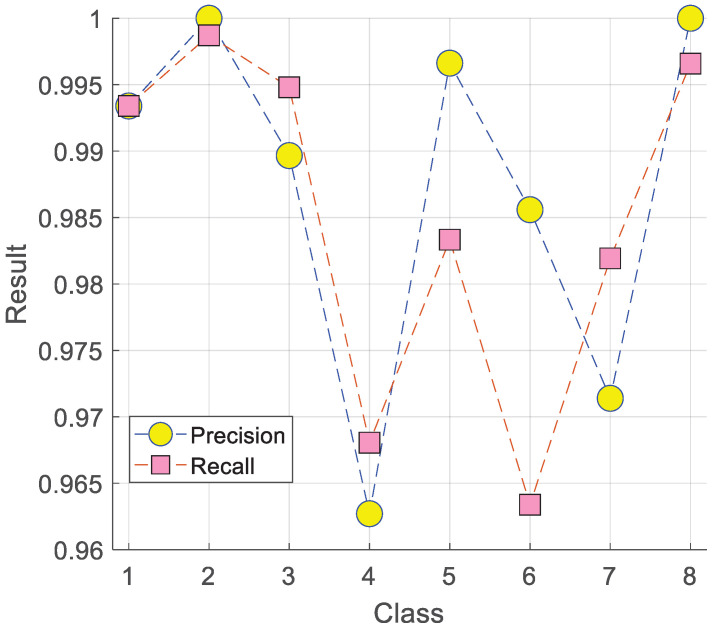
Test precision–recall (P–R) curves for the blood-cell image dataset.

Table 10 highlights that TurkerNeXtV2 exceeds 98% on every evaluated metric. Additionally, the precision–recall curves for each class are presented in Figure 9.

Curves were computed from model probabilities on the independent test set. Precision represents the proportion of correctly classified positive samples among all predicted positives, while recall denotes the proportion of correctly classified positive samples among all true positives. Each curve corresponds to one class, and higher curves indicate stronger detection across thresholds. Figure 9 presents the precision–recall results of TurkerNeXtV2 on the blood-cell dataset. These metrics complement accuracy by demonstrating both the reliability of predictions and the model’s ability to capture all relevant cases in medical image classification [53,54].

In the results, the second class achieved the highest performance, showing both precision and recall values close to 1.0, which indicates strong predictive reliability and sensitivity for this class. By contrast, the lowest precision was observed for the fourth class, meaning the model produced more false positives in this category. The lowest recall was obtained for the sixth class, suggesting that some true cases in this category were missed. These differences are likely due to intra-class variability and the visual similarity of certain cell types, a challenge reported in previous CNN and transformer-based studies on blood-cell image classification [55,56].

Overall, the P–R results confirm that TurkerNeXtV2 maintains consistently high precision and recall across most classes to illustrate its robustness in biomedical image analysis. The variation across classes also highlights the importance of using precision and recall together rather than relying only on accuracy, since these metrics provide a more detailed view of model strengths and limitations.

### 4.2. Benchmark

In this section, comparative results for Stable ImageNet-1k and the blood-cell image dataset are presented.

**Figure 10:**
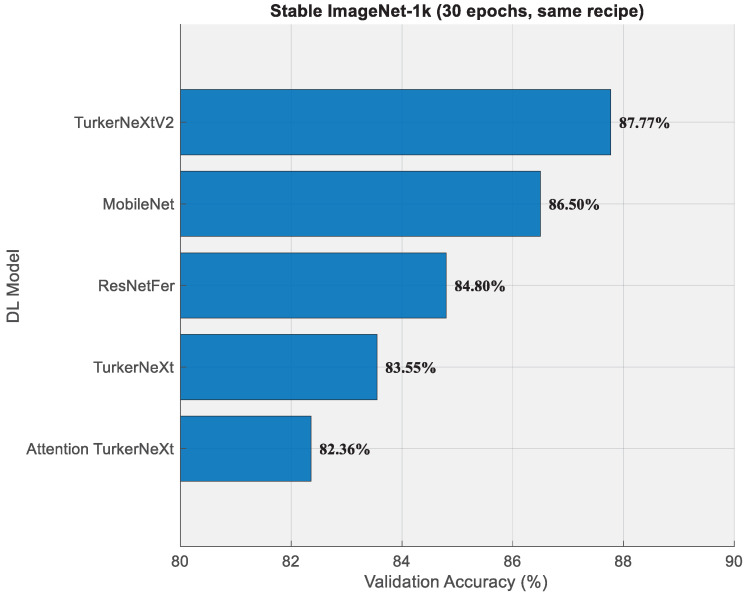
Comparative validation results on Stable ImageNet-1k. Shown are validation accuracies after 30 epochs for TurkerNeXtV2, MobileNet [51], ResNetFer [57], TurkerNeXt [45], and Attention TurkerNeXt [46]. All models were trained in MATLAB 2023a with the same settings: SGDM, batch size 128, initial learning rate 0.01, input 224 × 224. Computations were run on an RTX 5080 GPU. Values were obtained in-house, not from Kaggle. The figure highlights that, under identical conditions, TurkerNeXtV2 achieves the highest validation accuracy among lightweight CNN baselines and its predecessors.

Figure 10 openly shows that TurkerNeXtV2 achieves higher classification performance than competing models. MobileNet [51] and ResNetFer [57] were chosen because they represent widely used lightweight CNN baselines. MobileNet is known for its efficient depthwise separable convolutions [58]. Including these models ensures that TurkerNeXtV2 is compared against strong, efficient CNN references. The validation accuracies of other models were taken by Kaggle [59]. Moreover, we trained TurkerNeXt and Attention TurkerNeXt. Under a matched 30-epoch budget on Stable ImageNet-1k, TurkerNeXtV2 achieved 87.77% validation accuracy and outperformed MobileNet (86.50%), ResNetFer (84.80%), TurkerNeXt [45] (83.55%), and Attention TurkerNeXt [46] (82.36%). All models used 224 × 224 inputs, SGDM with 1 × 10^−2^ learning rate. For the blood-cell image dataset [14,15], comparative results are provided in Table 11.

Table 11 compares TurkerNeXtV2 with published CNN and transformer baselines (for example, Inception, VGG, MobileNet, ConcatNeXt, BloodCell-Net, MedViT, ViT, Swin). Accuracy values are reported as percent. Where a method targets four classes, the table indicates this explicitly. Results are taken from the cited sources or from re-implementations when specified in the text. Abbreviations: CNN, convolutional neural network; ViT, Vision Transformer; PR, precision–recall.

Table 11 demonstrates that the recommended TurkerNeXtV2 attains 98.52% accuracy and ranks second overall. The only higher score is a two-stage pipeline (ConcatNeXt + INCA + SVM, 98.73%). Among end-to-end models, TurkerNeXtV2 is the best or tied within the margin of error. Transformer baselines (Shifted-Window ViT 98.03%, MedViT 95.40%, Swin + ConvMixer 95.66%) trail our model; Big-Transfer (BiT) + Efficient-KAN reports 97.39%.

### 4.3. Key Points

We report the following: (i) architecture design (attention unit, downsampling, stem/head), (ii) model size (parameters), (iii) training recipe (MATLAB DND defaults: SGDM, LR 0.01, batch 128, 30 epochs, input 224 × 224), (iv) accuracy on Stable ImageNet-1k (validation), osteoarthritis pressure images (test), and the blood-cell dataset (test), (v) efficiency (latency and images/s on RTX 5080), and (vi) interpretability (Grad-CAM clarity).

TurkerNeXtV2 differs in three core ways that the table makes explicit:

TNV2 block: pooling-based attention fused with an inverted bottleneck (parameter-free attention + low-cost expand–compress) for stable, discriminative features.

Hybrid downsampling: max pool + average pool + grouped convolution to reduce routing bias while preserving salient pressure cues.

Simplified ConvNeXt stem and head: a 4 × 4, stride-4 patchify stem with a light inverted bottleneck and a GAP + FC head, which lowers activations and keeps the model compact.

Under the same training settings and hardware, TurkerNeXtV2 achieves higher or comparable accuracy to lightweight CNN baselines while remaining small (≈6.3–7.1 M parameters) and fast (≈0.0078 s/img; ≈128.8 images/s). It also provides clear Grad-CAM maps aligned with focal loading zones. Where prior work uses different class counts (for example, 4-class blood-cell variants), this is indicated in the table to keep comparisons fair.

In this research, we presented lightweight version of the presented TurkerNeXtV2. Lightweight means high accuracy with few parameters, and small activation memory, which directly affects latency, energy use, and deployability on routine clinic hardware, pressure-plate PCs, and wearable/edge devices. Storage space is part of this goal: TurkerNeXtV2 has 6.3–7.1 million parameters, which corresponds to roughly 25.3 MB in FP32 (4 bytes/parameter). The design also reduces RAM usage during inference by using an early 4 × 4/stride-4 stem (smaller feature maps), grouped/1 × 1 convolutions (fewer weights and activations), hybrid downsampling (faster spatial reduction without information loss), and a GAP + FC head (no large dense layers). Thus, the model is both computationally light and space-efficient. If tighter storage is required, standard compression can further shrink the footprint (e.g., FP16 quantization to ~12–14 MB, INT8 to ~6–8 MB with careful calibration), while preserving the low-compute advantages that enable real-time use in gait labs and point-of-care settings.

Findings:

- The presented CNN achieved 93.40% test accuracy on the osteoarthritis pressure-image dataset.
- TurkerNeXtV2 delivered 93.19% precision, 93.38% recall, and 93.28% F1-score on the same dataset.
- Our CNN reached 93.21% sensitivity, 93.54% specificity, and 93.37% geometric mean for osteoarthritis screening.
- TurkerNeXtV2 attained 87.77% validation accuracy on Stable ImageNet-1k after only 30 epochs.
- The recommended DL architecture maintained a lightweight footprint of 7.1 M parameters for 1000-class Stable ImageNet-1k and 6.3 M for 10-class osteoarthritis.
- TurkerNeXtV2 scored 98.52% test accuracy on the public blood-cell dataset, demonstrating strong cross-domain performance.
- 98.74% precision, 98.50% recall, and 98.62% F1-score were computed on the blood-cell dataset.
- TurkerNeXtV2 showcased best precision-recall on class 2 and lowest on classes 4 and 6 of the blood-cell task.
- Our model produced Grad-CAM heatmaps highlighting medial/lateral knee compartments in osteoarthritis and diffuse patterns in controls.
- TurkerNeXtV2 unified ConvNeXt-like stem, pooling-based attention, inverted bottleneck, and hybrid downsampling in one lightweight CNN.
- TurkerNeXtV2 demonstrated consistently high performance across medical (osteoarthritis and blood-cell) and general (Stable ImageNet-1k) domains.

Advantages:

- A new pressure-image dataset for osteoarthritis detection has been provided.
- Three new generation blocks have been utilized in this research to create the introduced TurkerNeXtV2
- High test accuracy (93.40%) is achieved on the osteoarthritis task.
- Strong generalization is demonstrated on Stable ImageNet-1k (87.77%) and a blood-cell dataset (98.52%).
- A lightweight architecture (~6–7 M parameters) is maintained.
- Transformer-inspired attention is implemented without self-attention, keeping computational cost low.
- Clinically interpretable Grad-CAM heatmaps are produced automatically.
- Modular blocks (stem, TNV2, downsampling, output) are easily scaled or adapted.

Limitations:

- The model has only been tested on two medical datasets and Stable ImageNet-1k; its robustness across other domains has not yet been examined.
- Our dataset is modest and from a single center, which may limit coverage of inter-subject variability and device bias. We did not use GAN-based data synthesis in this study.

Future directions:

- The TurkerNeXtV2 architecture will be extended to multi-class osteoarthritis grading tasks that distinguish early, moderate, and severe stages.
- We will evaluate GAN-based augmentation (e.g., CycleGAN/StyleGAN/diffusion) together with conventional augmentations (mixup, CutMix, RandAugment, color and geometric transforms, normalization/denoising) under label-preserving constraints to improve data quality and model generalizability.
- Lighter or larger versions of the TurkerNeXtV2 will be tested on the other image datasets.
- We will conduct shared-budget hyperparameter optimization (e.g., Bayesian optimization or multi-fidelity bandits) to improve TurkerNeXt’s classification performance across datasets.
- In TurkerNeXtV3, patch embedding and multi-head self-attention (MHSA) blocks are planned to be combined with pooling and convolution blocks.
- Lightweight mobile and web deployment versions will be developed for point-of-care screening devices.
- Federated-learning experiments will be conducted to train the model across multiple hospitals while preserving patient privacy.
- Additional ablation studies will be performed to quantify the individual contributions of the pooling-based attention and hybrid downsampling blocks.
- The model will be benchmarked on larger, publicly available biomedical datasets (e.g., chest X-ray, dermoscopy) to confirm cross-domain generalizability.

Potential applications:

- The recommended TurkerNeXtV2 is designed to run on existing pressure-plate systems and smart insoles used in gait labs and clinics, to provide real-time, interpretable decision support for medical professionals, and to complement standard imaging (X-ray or MRI) rather than replace it.
- Early OA risk assessment from plantar-pressure data acquired on standard pressure plates or smart insoles in clinics and sports centers; results support clinical judgment and may guide when to order X-ray or MRI.
- TurkerNeXtV2 can analyze pressure trials in real time, reports asymmetry and load indices, and sends summaries to hospital systems for clinician review.
- Remote and longitudinal monitoring with smart insoles or mats at home; data are uploaded securely for follow-up, rehabilitation tracking, and flare-up alerts set by clinicians.
- Pressure-based risk flags can be combined with other modalities in clinic.
- A large vision model (LVM) can be developed using larger version of the introduced TurkerNeXtV2.

## 5. Conclusions

This research introduced TurkerNeXtV2, a lightweight CNN with a compact architecture comprising 6.3–7.1 million parameters. The model achieved 87.77% validation accuracy on the Stable ImageNet-1k dataset, confirming its representational ability on a large-scale benchmark. When fine-tuned on specific domains, it reached 93.40% accuracy on osteoarthritis pressure images and 98.52% accuracy on blood-cell images. These results show that TurkerNeXtV2 is effective for both general image classification and specialized medical imaging tasks.

The strength of TurkerNeXtV2 comes from three design elements: a ConvNeXt-inspired stem, the TNV2 block that combines pooling-based attention with an inverted bottleneck, and a hybrid downsampling layer. Together, these components make the model compact, computationally efficient, and scalable, while attaining strong accuracy.

Because of its small size and pretrained weights, TurkerNeXtV2 can be adapted to new tasks with limited training and can be deployed in mobile applications, portable devices, and point-of-care diagnostic tools.

In future work, TurkerNeXtV2 should be tested on larger and multi-center medical datasets to confirm its generalizability, extended to different imaging modalities such as MRI, CT, and histopathology, and combined with explainable AI techniques to improve clinical trust. In addition, further studies should explore optimization for on-device deployment and energy-efficient inference so that the model can be used in real-time and resource-limited environments.

## Figures and Tables

**Table 1:** Summary of recent deep learning studies for knee osteoarthritis detection.

Study (Year)	Data/Modality	Model (Type)	Task	Performance (Metric)	Validation	Pitfalls/ Limitations
Tiulpin et al., 2018 [30]	MOST & OAI X-rays (~24k images)	Deep Siamese CNN ensemble	KL 0–4 grading	Acc 66.71%, AUC 0.93, κ 0.83	Yes (cross-dataset)	Early grades remain hard; dataset-specific biases.
Guan et al., 2022 [31]	OAI X-rays (6567 knees) + clinical	YOLO ROI + EfficientNet + ANN (fusion)	Pain progression (48 mo)	Combined AUC 0.807, Sens 72.3%, Spec 80.9%	Hold-out	Predicts pain (not structure); label noise risk.
Abdullah & Rajasekaran, 2022 [32]	Madurai X-rays (3172 images)	Faster-R-CNN (ROI) + ResNet-50 + AlexNet	ROI detect. + KL grading	ROI 98.52%, OA cls 98.90%	Hold-out	Potential leakage if not patient-wise; local dataset.
Wang et al., 2021 [33]	OAI X-rays (4.5k samples)	YOLO + Visual Transformer + ResNet50	Knee detection + KL grading	Detect 95.57%, KL acc 69.18%	Hold-out	Limited external test; moderate KL accuracy
Hu et al., 2023 [34]	MRI, FNIH cohort (*n* = 364; SAG-IW-TSE-FS, 3D-DESS)	3D DenseNet169	KOA progression (24–48 mo)	AUC 0.664 → 0.739 → 0.775 (baseline/12 mo/24 mo)	Multi-timepoint	Small cohort; MRI cost; heavy model.
Rani et al., 2024 [35]	OAI X-rays	12-layer CNN	Binary KOA + KL severity	Binary acc 92.3%, Multiclass acc 78.4%, F1 96.5%	Hold-out	Class imbalance; limited external test.
Ahmed & Imran, 2024 [36]	“Knee OA Severity Grading” X-rays (~8260; KL 0–4)	Fine-tuned CNNs (VGG/ResNet/EfficientNet-b7) + Grad-CAM	Binary & multi-class	Binary 99.13%, Multi-class 67%	Hold-out	Multi-class still weak; dataset imbalance.
Touahema et al., 2024 [37]	OAI (5k) + Medical Expert I/II + local	Pre-trained Xception (TL); GUI tool	KL grading/severity	Val 99.39%, Test 97.20%, External ≈ 95%	Some external sets	Possible overlap; unclear patient-wise split; very high scores need replication.
Lee et al., 2024 [38]	OAI → MOST (independent test 17,040 images)	Plug-in modules compatible with CNN/Transformer	KL 0–4 grading	Per-grade acc: KL1 43%, KL4 96%	Yes (OAI→MOST)	KL-1 hard; added complexity.
Sekhri et al., 2023 [16]	OAI (+public sets) X-rays	Swin Transformer variants	KL 0–4 grading	Acc ~70%, F1 ~0.67 (reported)	Partial	In many cases; details/splits vary.
Abdullah et al., 2025 [39]	4334 digital knee X-rays (AP and lateral views)	Faster R-CNN for JSW localization + Fine-tuned DenseNet-201 for KL grading	Localization of tibio-femoral joint space (JSW) + KL classification (1–4) from AP & Lat views	Localization acc: AP 98.74%, Lat 96.12% KL classification acc: AP 98.51%, Lat 92.42% Kappa: AP 0.98, Lat 0.91	Hold-out	Lateral view underperforms due to overlapping anatomy Longer training times due to multi-stage pipeline
Vaattovaara et al., 2025 [40]	208 AP knee X-rays (external test set) Train: MOST Val: OAI	Deep Siamese CNN + Hourglass knee localization	KL grading (0–4) multi-class & binary (0–1 vs. 2–4)	Multi-class AUC: 0.886 Balanced Acc: 69.3% Kappa: 0.820 Binary AUC: 0.967 Balanced Acc: 90.2%	External test set (*n* = 208), comparison with 4 readers	KL1 sensitivity low (37.2%) Grade imbalance DL underperforms vs. expert MSK radiologists
Yayli et al., 2025 [41]	Knee X-ray (AP), 14,607 images, 3 hospitals	Single-model CNNs (EfficientNet, NfNet, etc.) vs. multi-model pipeline	KL staging	Best single-model: F1 = 0.763, Acc = 0.767; multi-model lower (F1 = 0.736, Acc = 0.740)	Hold-out	CLAHE often hurt performance; class imbalance
Bai et al., 2025 [42]	Plantar pressure (RSscan plate), *n* = 243 healthy males	PCA + optimized K-means + LDA (classical ML)	Classifying plantar pressure distribution types	Acc (original) 89.70%; cross-validated Acc 88.50% (3 classes)	10-fold CV	Healthy young males only; not disease labels; ML pipeline depends on hand-crafted indices
Ma’aitah et al., 2025 [43]	OAI knee X-rays + text	Multimodal Transformer (ViT for image + BERT for text)	KL grading (multimodal)	Overall Acc = 82.85%; Precision = 84.54%; Recall = 82.89	Hold-out	Evaluation only on OAI; dependency on text description
Abdusalomov et al., 2025 [44]	“KOA Severity Grading” X-ray dataset; athletes focus	Lightweight CNN (EfficientNet-B0 + ECA	Early-stage vs. healthy (binary)	Accuracy ≈ 92% (range 91.5–92% in the report)	Hold-out	Binary task; KL 0–4 generalizability to full rating uncertain
Tuncer et al., 2023 [45]	Chest X-ray; 4 class: heart failure, Coah, COVID-19, healthy	Lightweight CNN (≈5.7 M parameters); attention + ConvNeXt block fusion; additionally, TurkerNeXt-based deep feature extraction + SVM classifier	Multi-class disease classification (4-class)	Training accuracy 100%; validation accuracy 90.19%; TurkerNeXt-based deep feature engineering model test accuracy 97.36%	Hold-out	Limited data/cohort details
Arslan et al., 2023 [46]	OCT retina; top-to-bottom/left-to-right B-scan cases and merged	Attention CNN (~1.6 M parameters); patchify stem + Attention TurkerNeXt blocks; ConvNeXt/Swin/MLP/ResNet-inspired architecture	Binary classification: bipolar disorder vs. healthy	Validation and testing in all cases = 100% (accuracy, sensitivity, specificity, precision, F1, G-mean)	Hold-out	The need for larger and more diverse OCT datasets

* CNN: Convolutional Neural Network; DL: Deep Learning; ANN: Artificial Neural Network; ROI: Region of Interest; KL: Kellgren–Lawrence; AUC: Area Under Curve; MRI: Magnetic Resonance Imaging; GUI: Graphical User Interface; Grad-CAM: Gradient-weighted Class Activation Mapping; SVM: Support Vector Machine; OCT: Optical Coherence Tomography; PCA: Principal Component Analysis; LDA: Linear Discriminant Analysis; Acc: Accuracy; κ: Cohen’s Kappa; Sens: Sensitivity; Spec: Specificity; F1: F1-Score; CV: Cross-Validation; 10-fold CV: Ten-Fold Cross-Validation; Hold-out: Single Fixed Split; External test: Test Set from a Different Cohort/Site; JSW: Joint-Space Width; AP: Anteroposterior; Lat: Lateral; MSK: Musculoskeletal; ViT: Vision Transformer; ECA: Efficient Channel Attention; BERT: Bidirectional Encoder Representations from Transformers; CLAHE: Contrast-Limited Adaptive Histogram Equalization; RSscan: Plantar Pressure Plate System; SAG-IW-TSE-FS: Sagittal Intermediate-Weighted Turbo Spin-Echo with Fat Suppression; 3D-DESS: Three-Dimensional Double-Echo Steady-State.

**Table 2:** Demographic and clinical characteristics of the healthy and osteoarthritis group.

Variable	Healthy Group (KL 0)	OA Group (KL 4)
Age (years) (Mean ± SD)	56.42 ± 6.21	65.27 ± 3.53
Sex	Female: 51 (56.7%)/Male: 39 (43.3%)	Female: 42 (77.8%)/Male: 12 (22.2%)
Height (cm) (Mean ± SD)	166.6 ± 7.24	159.1 ± 4.77
BMI (kg/m^2^) (Mean ± SD)	23.9 ± 2.87	26.5 ± 5.10

* cm: Centimeter; OA: Osteoarthritis; SD: Standard Deviation; BMI: Body Mass Index; kg/m^2^: kilograms per square meter.

**Table 3:** Distribution of the collected plantar-pressure images.

No	Class	Training (Images)	Test (Images)	Total (Images)
1	Osteoarthritis	888	221	1109
2	Control	1180	294	1474
Total		2068	515	2583

**Table 4:** TurkerNeXtV2 stage transitions and tensor shapes.

Stage	Input	Operation	Output
**Stem**	224 × 224 × 3	$\left[\begin{array}{c} 4\times 4,\;96,\;St=4\\ 1\times 1,\;384\\ 1\times 1,\;96 \end{array}\right]$	56 × 56 × 96
**TNV2 1**	56 × 56 × 96	$\left[\begin{array}{c} {(P}_{3\times 3}^{A},96)\times {(P}_{3\times 3}^{M},96)\\ P_{3\times 3}^{A},192\\ 1\times 1,\;96\;\;\\ 1\times 1,\;384\\ 1\times 1,\;96 \end{array}\right]\times 1$	56 × 56 × 96
**Downsampling 1**	56 × 56 × 96	$DC({(P}_{2\times 2,St=2}^{A},96),{(P}_{2\times 2,St=2}^{M},96))$ 2×2, 192, St=2	28 × 28 × 192
**TNV2 2**	28 × 28 × 192	$\left[\begin{array}{c} {(P}_{3\times 3}^{A},192)\times {(P}_{3\times 3}^{A},192)\\ P_{3\times 3}^{A},384\\ 1\times 1,\;192\;\;\\ 1\times 1,\;768\\ 1\times 1,\;192 \end{array}\right]\times 1$	28 × 28 × 192
**Downsampling 2**	28 × 28 × 192	$DC({(P}_{2\times 2,St=2}^{A},192),{(P}_{2\times 2,St=2}^{A},192))$ 2×2, 384, St=2	14 × 14 × 384
**TNV2 3**	14 × 14 × 384	$\left[\begin{array}{c} {(P}_{3\times 3}^{A},384)\times {(P}_{3\times 3}^{A},384)\\ P_{3\times 3}^{A},768\\ 1\times 1,\;384\;\;\\ 1\times 1,\;1536\\ 1\times 1,\;384 \end{array}\right]\times 1$	14 × 14 × 384
**Downsampling 3**	14 × 14 × 384	$DC({(P}_{2\times 2,St=2}^{A},384),{(P}_{2\times 2,St=2}^{M},384))$ 2×2, 768, St=2	7 × 7 × 768
**TNV2 4**	7 × 7 × 768	$\left[\begin{array}{c} {(P}_{3\times 3}^{A},768)\times {(P}_{3\times 3}^{M},768)\\ P_{3\times 3}^{A},1536\\ 1\times 1,\;768\;\;\\ 1\times 1,\;3072\\ 1\times 1,\;768 \end{array}\right]\times 1$	7 × 7 × 768
**Output size**	7 × 7 × 768	GAP, FC, Softmax	Number of classes
**Total learnable parameters**			For 10 classes ~6.3 Million For 10,000 classes ~7.1 Million

* $DC\left(.\right):$ Depth Concatenation, $P^{A}(.)$: Average Pooling, $P^{M}(.)$: Maximum Pooling, St: Stride.

**Table 5:** Training and validation results on Stable ImageNet-1k.

Training Loss	0.0331
Training Accuracy	100%
Validation Loss	0.4647
Validation Accuracy	87.77%

**Table 6:** Validation results after domain adaptation of TurkerNeXtV2 on the osteoarthritis pressure-image dataset.

Training Loss	1.52 × 10^−6^
Training Accuracy	100%
Validation Loss	0.3168
Validation Accuracy	93.72%

**Table 7:** Test results on the osteoarthritis pressure-image dataset.

Performance Assessment Metrics	Class	Results (%)
Accuracy	Overall	93.40
Precision	Osteoarthritis	91.56
	Control	94.83
	Overall	93.19
Recall	Osteoarthritis	93.21
	Control	93.54
	Overall	93.38
F1-score	Osteoarthritis	92.38
	Control	94.18
	Overall	93.28

**Table 8:** Comparative inference efficiency of TurkerNeXtV2 and baseline models on RTX 5080 hardware using MATLAB implementation.

Model	Avg. Time (s)	Images/s	Relative to TurkerNeXtV2
TurkerNeXtV2 (ours)	0.0078	128.81	–
ResNet50	0.0078	128.38	≈Same (−0.3%)
GoogLeNet	0.0070	141.94	Faster (+10.2%)
MobileNetV2	0.0095	105.64	Slower (−18.0%)
DenseNet201	0.0422	23.71	Much slower
EfficientNetB0	0.0349	28.62	Much slower
NasNetMobile	0.0521	19.21	Much slower
InceptionV3	0.0138	72.32	Slower (−43.9%)
InceptionResNetV2	0.0429	23.29	Much slower
DarkNet53	0.0123	81.04	Slower (−37.1%)
Vision Transformer (ViT)	0.0217	46.12	Much slower

Large negative deviations (>|50%|) were categorized as much slower.

**Table 9:** Training and validation results of the TurkerNeXtV2 on the blood image dataset.

Training Loss	2.29 × 10^−5^
Training Accuracy	100%
Validation Loss	0.0571
Validation Accuracy	98.95%

**Table 10:** Test results on the blood-cell image dataset.

Performance Assessment Metrics	Results (%)
Accuracy	98.52
Precision	98.74
Recall	98.50
F1-score	98.62

**Table 11:** Comparative results on the blood-cell image dataset.

Study	Method	Accuracy (%)
Acevedo et al. [14]	Inceptionv3 + Softmax	94.90
Acevedo et al. [14]	VGG16 + Softmax	96.20
Acevedo et al. [14]	Inceptionv3 + SVM	90.50
Acevedo et al. [14]	VGG16 + SVM	87.40
Dwivedi and Dutta [60]	Microcell-Net	97.65
Ammar et al. [61]	CNN + Adaboost	88.80
Erten et al. [62]	ConcatNeXt + Softmax	97.77
Erten et al. [62]	ConcatNeXt + INCA + SVM	98.73
Mondal et al. [63]	BloodCell-Net	97.10
Rastogi et al. [64]	LeuFeatx + SVM	91.62
Khan et al. [65]	DCGAN + MobileNetV2	93.83
Bilal et al. [66]	ConvLSTM	96.90
Manzari et al. [67]	MedViT	95.40
Chen et al. [55]	Shifted Window ViT (for four classes)	98.03
Kokcam and Ucar [68]	Big Transfer (BiT) and Efficient KAN	97.39
Uzen and Firat [56]	Swin transformer and ConvMixer (for four classes)	95.66
**This Paper**	TurkerNeXtV2	98.52

## Data Availability

The data presented in this study are available on request from the corresponding author due to privacy restrictions and the use of private data.

## Abbreviations

**Clinical, imaging & study terms**	
**AP**	Anteroposterior (X-ray view)
**B-scan**	Cross-sectional OCT scan
**BMI**	Body Mass Index
**JSW**	Joint Space Width
**KL**	Kellgren–Lawrence grading
**KOA**	Knee Osteoarthritis
**Lat**	Lateral (X-ray view)
**mo**	Months (time horizon)
**MRI**	Magnetic Resonance Imaging
**MSK**	Musculoskeletal (radiologists)
**OA**	Osteoarthritis
**OCT**	Optical Coherence Tomography
**SAG-IW-TSE-FS**	Sagittal Intermediate-Weighted Turbo Spin-Echo with Fat Saturation (MRI sequence)
**SD**	Standard Deviation
**3D-DESS**	Three-Dimensional Double-Echo Steady-State (MRI sequence).
**Datasets & initiatives**	
**OAI**	Osteoarthritis Initiative.
**MOST**	Multicenter Osteoarthritis Study.
**FNIH**	Foundation for the National Institutes of Health (cohort/consortium context).
**PROGRESS OA**	FNIH Biomarkers Consortium PROGRESS OA study.
**Biomarkers & wearable/gait systems**	
**uCTX-II/sCTX-II**	Urinary/Serum C-terminal telopeptide of type II collagen.
**COMP**	Cartilage Oligomeric Matrix Protein.
**PIIANP**	Procollagen II N-terminal propeptide.
**T2, T1ρ, dGEMRIC**	Quantitative MRI markers.
**IMU**	Inertial Measurement Unit (wearable).
**GAITRite**	Instrumented walkway system (brand).
**sCTX-II**	Serum C-terminal telopeptide of type II collagen
**Methods, architectures & components**	
**ANN**	Artificial Neural Network
**BERT**	Bidirectional Encoder Representations from Transformers
**CNN**	Convolutional Neural Network
**CLAHE**	Contrast-Limited Adaptive Histogram Equalization
**ConvNeXt**	ConvNeXt architecture (patchify stem)
**DC**	Depth Concatenation
**DL**	Deep Learning
**ECA**	Efficient Channel Attention
**EfficientNet**	EfficientNet CNN family
**FC**	Fully Connected layer
**Faster R-CNN**	Region-based CNN detector
**GAP**	Global Average Pooling
**GELU**	Gaussian Error Linear Unit (activation)
**Grad-CAM**	Gradient-weighted Class Activation Mapping
**GUI**	Graphical User Interface
**LDA**	Linear Discriminant Analysis
**ML**	Machine Learning
**MLP**	Multi-Layer Perceptron
**NfNet**	Normalizer-Free Network
**PoolFormer**	PoolFormer architecture
**ResNet**	Residual Network
**ROI**	Region of Interest
**SVM**	Support Vector Machine
**TL**	Transfer Learning
**Transformer (ViT, Swin)**	Vision Transformer/Swin Transformer
**TNV2**	TurkerNeXtV2 block
**U-Net**	U-Net segmentation network
**XAI**	Explainable Artificial Intelligence
**Xception**	Xception CNN (pretrained)
**YOLO**	“You Only Look Once” object detector.
**Evaluation metrics**	
**Acc**	Accuracy.
**AUC**	Area Under the (ROC) Curve.
**F1/F1-score**	F1 measure.
**κ (Kappa)**	Cohen’s Kappa agreement.
**Sens**	Sensitivity (Recall).
**Spec**	Specificity.
**Balanced Acc**	Balanced Accuracy.
**G-mean**	Geometric Mean.
**Val**	Validation (set/score).
**Imaging views & protocol notes**	
**AP**	Anteroposterior (X-ray view).
**Lat**	Lateral (X-ray view).
**Training & compute**	
**SGDM**	Stochastic Gradient Descent with Momentum.
**LR**	Learning Rate.
**L2**	L2 regularization (weight decay).
**PC**	Personal Computer.
**GPU**	Graphics Processing Unit.
**RTX**	NVIDIA RTX (graphics card series).
**FLOPs**	Floating-Point Operations.
**FP32/FP16/INT8**	32-bit/16-bit floating-point; 8-bit integer (quantization).
**RAM**	Random Access Memory.
